# A multi-objective scheduling method for operational coordination time using improved triangular fuzzy number representation

**DOI:** 10.1371/journal.pone.0252293

**Published:** 2021-06-09

**Authors:** Luda Zhao, Bin Wang, Congyong Shen

**Affiliations:** 1 College of Electronic Engineering, National University of Defense Technology, Hefei, China; 2 Third Interdisciplinary Center (HeFei), National University of Defense Technology, Hefei, China; University of Defence in Belgrade, SERBIA

## Abstract

In modern warfare, the comprehensiveness of combat domain and the complexity of tasks pose great challenges to operational coordination.To address this challenge, we use the improved triangular fuzzy number to express the combat mission time, first present a new multi-objective operational cooperative time scheduling model that takes the fluctuation of combat coordinative time and the time flexibility between each task into account. The resulting model is essentially a large-scale multi-objective combinatorial optimization problem, intractably complicated to solve optimally. We next propose multi-objective improved Bat algorithm based on angle decomposition (MOIBA/AD) to quickly identify high-quality solutions to the model. Our proposed algorithm improves the decomposition strategy by replacing the planar space with the angle space, which helps greatly reduce the difficulty of processing evolutionary individuals and hence the time complexity of the multi-objective evolutionary algorithm based on decomposition (MOEA/D). Moreover, the population replacement strategy is enhanced utilizing the improved bat algorithm, which helps evolutionary individuals avoid getting trapped in local optima. Computational experiments on multi-objective operational cooperative time scheduling (MOOCTS) problems of different scales demonstrate the superiority of our proposed method over four state-of-the-art multi-objective evolutionary algorithms (MOEAs), including multi-objective bat Algorithm (MOBA), MOEA/D, non-dominated sorting genetic algorithm version II (NSGA-II) and multi-objective particle swarm optimization algorithm (MOPSO). Our proposed method performs better in terms of four performance criteria, producing solutions of higher quality while keeping a better distribution of the Pareto solution set.

## 1 Introduction

Operational coordination refers to the coordination and cooperation among various combat forces in actions according to a unified plan when they jointly perform combat tasks, so as to ensure the coordinated actions of various combat forces and exert their overall combat effectiveness [1]. With the development of miniaturization and intelligence of the war, it is of great significance to study the combat coordination in tactical operations for improving the combat capability of the troops.

In the field of multi-agent model optimization and network task optimization, the problem of combat task scheduling has been widely studied. Multi-agent-based task scheduling [2, 3] makes rational planning for task subject through optimization algorithm, and obtains the reasonable task scheduling result. Task scheduling based on time network [4, 5] connects different task time nodes into a directed network, and obtains a reasonable task timing scheduling result by optimizing the parameters of the nodes and the whole network. Although there are many solutions to the time series planning of combat coordination, under the above-mentioned operational background, we can draw the conclusion that solving the time problem of combat coordination in Observe-Orient-Decide-Act (OODA) loop mainly has the following challenges.

1. When solving the operational coordination time sequence, how to fully consider the complex factors affecting operations, and reasonably model the execution time and completion time series of each operational task based on the complex factors, so as to make it closer to the actual combat;
2. When considering the execution time of all combat tasks, how to better consider the execution time of each combat task, and whether the solved task time and combat sequence are still valid and the robustness of the model is good enough under the sudden change of battlefield situation;
3. When solving the established model, how to choose a suitable algorithm to solve the model quickly and effectively.

The purpose of this paper aims to solve the problem of cooperation time and cooperation time sequence in complex combat. The major innovation and contributions of this paper can be summarized as follows. Firstly, considering the complexity of battle, a MOOCTS model is established. In this model, the improved triangular fuzzy number is used to re-describe the combat mission time, and the ranking function of the triangular fuzzy number and the calculation method of the probability degree are given, and the flexible combat time among various combat missions is considered. Secondly, the approach based on angle space decomposition improves the aggregation method of the traditional MOEA/D, and introduces the solution space optimization strategy of the traditional bat algorithm (BA), proposes an improved MOBA. Finally, the improved MOEA is used to solve the MOOCTS model, and several common MOEAs are used to solve this model. The performance and robustness of the algorithms are compared and discussed.

The outline of the paper is as follows: Section 2 will briefly review previous work. In Section 3, a MOOCTS model is established. Section 4 presents the proposed solution algorithm, MOIBA/AD. Some numerical experiment is carried in Section 5. The conclusion and future work are presented in Section 6.

## 2 Related work

### 2.1 Operational task time sequence cooperative scheduling

At present, when studying the scheduling of combat coordination, there are mainly two ways of thinking. One is based on the task chain formed by different tasks, which takes the completion cost, completion efficiency and resource utilization efficiency of combat tasks as the optimization objectives, and carries out collaborative planning through the time sequence relationship and resource constraints in the task chain, thus obtaining the overall time scheduling strategy. For example, Zhou et al. [6] took the operational time and operational resource utilization as the cooperative optimization objectives, the role-based artificial bee colony (RABC) algorithm is used to calculate the cooperative time plan. Wang et al. [7] took the operational timeliness as their optimization objective, and based on considering the constraints such as task timing, task completion effect and task execution conflict, they used the invasive weed bat and twin group optimization (IWBDA) method to plan and solve the Operational task time sequence cooperative scheduling problem. Cai et al. [8] used multi-dimensional asynchrony and inertia weight to improve the particle swarm optimization algorithm (PSO), the problem of timing relationship planning in the command and control tasks is solved. Li et al. [9] considered the priorities of different combat tasks based on the traditional collaborative planning problem, and planned the task sequence according to different priorities, which was more in line with the actual combat. In addition, based on considering the priority of tasks and resources at the same time, many scholars use greedy strategy to locally search for the solution space of the multi-imensional dynamic list scheduling model (MDLS) [10–12] and multi-PRI list dynamic scheduling model (MPLDS) [13–15], which have achieved good results in the scheduling problem of cooperative task time. However, this method does not take into account the coupling between the execution subjects and time of different and the same combat tasks in the solving process. It basically believes that similar combat times will not cause conflicts between the same task subjects. But it is obviously not applicable to simply consider the time conflicts among various task chains in actual combat situations.

Another is to combine the combat processes with the task nodes and task arcs to form a simple time network (STN) [16] or a temporal constraint network (TCN) [17], which converts the task timing relationship solving into a network time conflict detection problem. When solving this kind of problems, Tang et al. [18] modeled the combat task time series using STN, and then resolved the conflicts in the time series network using constraint relaxation. Hunsberger et al. [19] improved the traditional STN network using dynamic consistency (DC) and obtained the conditional simple temporary networks (CSTNS), which further improved the time detection performance of the traditional network. Based on STN model, Ding et al. [20] adjusted the task priority and constraint in the model to solve the problem of time conflict. Jia et al. [21] and Huntzberg et al. [22] respectively modeled the time constraints of multi-level joint tasks and multi-unmanned submarine cooperative combat tasks using TCN, and successfully detected and resolved the time conflicts of the tasks. Chen et al. [23] simplified TCN to a certain extent, and let the attack time combined with the weight matrix method by using the simple time constraint network (STCN) according to the combat mode of the air force on the shore, which successfully detected the time consistency and solves the conflict of combat time. Qi et al. [24] further expanded TCN, and considering the complexity of large-scale task time planning, proposed a Hierarchical Task Network (HTN) based on hierarchical partition, which solved the problem well. In the second way of solving this kind of problem, the relationship between actual combat mission is too simple. Many arm of the services have mutual cooperation and support relations, the simple task chain of a single arm basically does not exist.

### 2.2 MOEAs for operational task scheduling

In the field of solving the problem of combat task scheduling, when the first idea in Section 2.1 is used to model, and the model objective is two or more, the multi-objective optimization method is needed to solve the model. Wu et al. [25] modeled the task scheduling with the preference of decision makers, and used improved non-dominated sorting genetic algorithm version III (NSGA-III) with knee points to effectively solve the problem. Ramezani et al. [26] established a multi-task sequential scheduling model in a distributed computing environment, and two multi-objective optimization algorithms, multi-objective genetic algorithm (MOGA) and MOPSO, are used to compare the model solution and algorithm performance. The results show that the algorithm performance of MOPSO is superior to that of MOGA in solving such problems. Cao et al. [27] and Liu et al. [28] made reasonable assumptions for multi-task scheduling in a dynamic environment and established a sequential scheduling model. By using MOEA/D method, the sequential scheduling scheme of tasks was obtained. In addition, NSGA-II [29, 30], strength Pareto evolutionary algorithm version II (SPEA-II) [31] and multi-objective artificial bee colony (MOABC) [32] are also used to solve multi-task scheduling. At present, the application of various multi-objective optimization algorithms in this kind of problems is still a very hot field, which is worth further study.

In addition, it is one of the widely used methods to evaluate and rank combat tasks by using various evaluation methods. For example, an evaluation method combining the Analytical Hierarchy Process (AHP) method and fuzzy mathematics [33–35], Technique for Order Preference by Similarity to an Ideal Solution (TOPSIS) method in dynamic environment [36–38], Data Envelopment Analysis (DEA) method which is less affected by the subjective factors of decision makers [39–41], and entropy method combined with the above method [42, 43], etc.

## 3 MOCCTS

**Fig 1:**
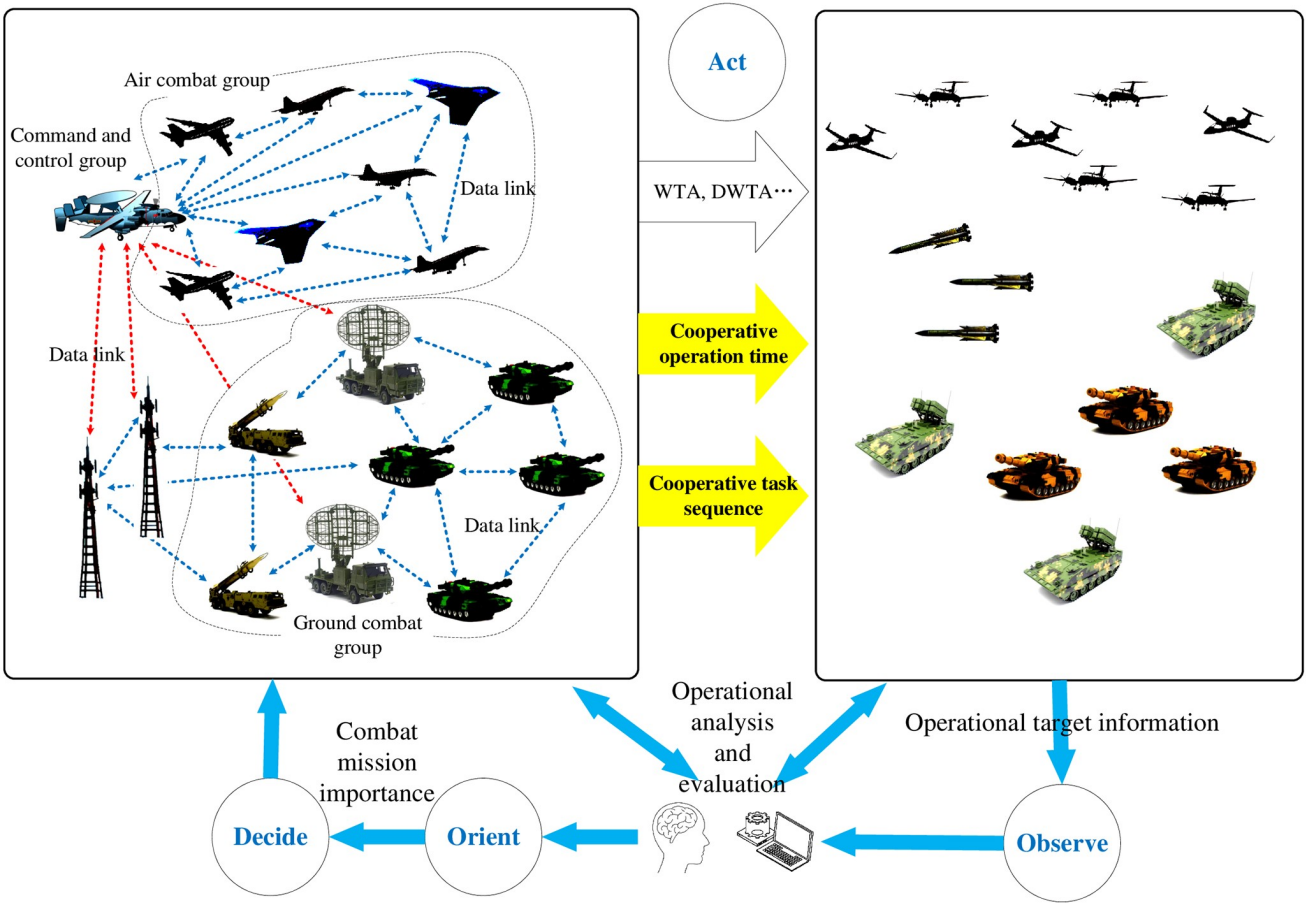
An illustration of operational coordination content in OODA loop.

Generally, a combat mission requires a complete OODA loop, as shown in Fig 1. Taking a joint land-air combat operation as an example, the combat weapons under the jurisdiction of our army can be divided into three clusters, namely, one land-air combat cluster, one air combat cluster and one command and control support cluster. Each cluster is internally connected through a communication data link (dotted blue line in the figure), and the clusters are connected together through sensors and data links (dotted red line in the figure). When carrying out tasks against operational targets, the three clusters conduct unified operations, such as reconnaissance, surveillance, and strikes. We continuously observe the combat targets through various investigation means to obtain detailed information of the combat targets, and feed back the information to a decision module consisting of a command decision maker and an auxiliary decision system, then the decision module comprehensively analyzes and judges the battlefield situation and the combat target information, obtains the importance level of each combat task faced by us, and further makes decisions on combat operations. The contents of combat decision-making generally includes the assignment of weapon target at each combat time, assignment of weapon target changing with dynamic time, duration of each combat task, coordinative time sequence of combat task and so on. This paper mainly studies the solution of the latter two problems, which are represented by yellow arrows in the figure.

### 3.1 Model assumptions and notation descriptions

The notation description of this model is shown in Table 1.

**Table 1:** Notation declaration.

Notation	Description
*I*	A set of sequence numbers for a combat mission, *I* = {1, 2, ⋯, *n*}, *i*, *i*′, *i*″ ∈ *I*;
*J*	A set of sequence numbers for the combat phase, *J* = {1, 2, ⋯, *l*}, *j*, *j*′, *j*″ ∈ *J*;
*K*	The sequence number set of the combat cluster, *K* = {1, 2, ⋯, *m*}, *k*, *k*′, *k*″ ∈ *K*;
*S*_*ij*_, *S*_*i*′*j*′_, *S*_*i*″*j*″_	A set of combat tasks that need to be completed in different combat phases;
$PT_{ij}^{k}$	Duration for combat cluster *k* to complete combat mission *i* in phase *j*;
$CT_{ij}^{k}$	Combat cluster *k* completes the end time of combat mission *i* in stage *j*;
*N*_*i*_	Number of combat clusters required at least to complete combat mission *i*;
*C*_*k*_	Combat consumption per unit time generated by combat cluster *k*;
*I*_*ij*_	Mission *i* importance factor in combat phase *j*, *I*_*ij*_ ∈ [0, 1];
$R_{j^{\prime },j^{\prime \prime }}^{i^{\prime },i^{\prime \prime }}$	Sequencing variable of combat mission, $R_{j^{\prime },j^{\prime \prime }}^{i^{\prime },i^{\prime \prime }}=1$ indicates that task *i*′ in combat phase *j*′ occurs before task *i*′ in combat phase *j*′; otherwise, $R_{j^{\prime },j^{\prime \prime }}^{i^{\prime },i^{\prime \prime }}=0$;
$q_{ij}^{k}$	Correspondence variable between combat cluster and combat task, $q_{ij}^{k}=1$ indicates that task *i* in operation phase *j* is completed by operation cluster *k*; otherwise, $q_{ij}^{k}=0$.

In order to make the model established in this paper closer to the actual combat and convenient for solving the model, the following explanations and assumptions are put forward.

1. In this model, combat clusters are regarded as the basic unit of operational coordination. Generally speaking, the task coordination scheme is to plan the task order for different specialties and combat clusters. When executing a specific task, the selection of the internal strength of the cluster is selected by the cluster commander according to the actual situation.
2. Each combat cluster adopts the “Start-End” mechanism when executing combat tasks, that is, once a combat task starts to execute, the clusters that complete the task must execute the current task to the end, and the execution of the current task can not be interrupted.
3. The importance ranking of all combat missions is known prior to model solving.
4. Each combat cluster can only carry out one combat task simultaneously in the same combat phase.
5. The occurrence of our equipment failure during combat is not considered.

### 3.2 Model formulation

#### 3.2.1 Objectives

This paper mainly considers the following two optimization objectives, that is, the maximum completion time of all tasks and the cost-effectiveness ratio of the whole combat operation.

The maximum completion time of all tasks refers to the end time of the last task in the whole operational process. We express it by the longest duration in all combat phases when *k* combat cluster performs *i* combat mission. Expressed as follows.

$$\begin{array}{c} F_{1}=\max\{\sum_{j=1}^{l}PT_{ij}^{k}\},\forall i\in I,k\in K \end{array}$$ (1)

The cost-effectiveness ratio of the whole combat operation refers to the ratio of combat effectiveness to combat consumption in the course of combat. Because the whole combat mission coordination time network is a time series directed graph composed of several subtasks, in the classical network analysis method, the “entropy” is generally used to measure the influence and coordination degree of the network system on the subsystems [44]. Cost-effectiveness ratio is one of the performance indexes of the whole cooperative time series network, and we also use the entropy value to express the traditional cost-effectiveness ratio. So, the combat effectiveness is expressed by the important entropy of the combat mission.

$$\begin{array}{c} Entropy_{impor\tan ce}=-\sum_{k=1}^{m}(\sum_{i=1}^{n}\sum_{j=1}^{l}I_{ij}q_{ij}^{k}PT_{ij}^{k}\ln(I_{ij}q_{ij}^{k}PT_{ij}^{k})) \end{array}$$ (2)

where $I_{ij}q_{ij}^{k}PT_{ij}^{k}$ indicates the importance of combat cluster *k* completing task *i* in phase *j*. Obviously, the smaller the value, the higher the combat effectiveness, and the larger the value, the lower the combat effectiveness. Therefore, in practice, we take the opposite number. Then, Considering the combat consumption *C*_*k*_ together with the combat importance entropy defined above, the cost-effectiveness ratio of the whole operation is expressed as follows.

$$\begin{array}{c} F_{2}=\frac{-Entropy_{impor\tan ce}}{\sum_{i=1}^{n}\sum_{j=1}^{l}\sum_{k=1}^{m}PT_{ij}^{k}q_{ij}^{k}C_{k}} \end{array}$$ (3)

The denominator in the above formula represents the sum of our combat consumption in the whole combat stage.

#### 3.2.2 Constraints


$$\begin{array}{c} \sum_{i=1}^{n}q_{ij}^{k}=1,\forall k\in K,j\in J \end{array}$$ (4)

$$\begin{array}{c} \begin{array}{cc} \left(1-R_{j^{\prime },j^{\prime \prime }}^{i^{\prime },i^{\prime \prime }})(CT_{i^{\prime }j^{\prime }}^{k}q_{i^{\prime }j^{\prime }}^{k}-CT_{i^{\prime \prime }j^{\prime \prime }}^{k}q_{i^{\prime \prime }j^{\prime \prime }}^{k}\right) & \geq (1-R_{j^{\prime },j^{\prime \prime }}^{i^{\prime },i^{\prime \prime }})PT_{i^{\prime }j^{\prime }}^{k},\\ & k\in (S_{i^{\prime }j^{\prime }}\cap S_{i^{\prime \prime }j^{\prime \prime }}),\forall i^{\prime },i^{\prime \prime }\in I,j^{\prime },j^{\prime \prime }\in J \end{array} \end{array}$$ (5)

$$\begin{array}{c} \begin{array}{cc} \left(1-R_{j^{\prime },j^{\prime \prime }}^{i^{\prime },i^{\prime }})(CT_{i^{\prime }j^{\prime }}^{k^{\prime }}q_{i^{\prime }j^{\prime }}^{k^{\prime }}-CT_{i^{\prime \prime }j^{\prime \prime }}^{k^{\prime \prime }}q_{i^{\prime \prime }j^{\prime \prime }}^{k^{\prime \prime }}\right) & \geq (1-R_{j^{\prime },j^{\prime \prime }}^{i^{\prime },i^{\prime }})PT_{i^{\prime }j^{\prime }}^{k^{\prime }},\\ & k^{\prime }\in S_{i^{\prime }j^{\prime }},k^{\prime \prime }\in S_{i^{\prime \prime }j^{\prime \prime }},\forall i^{\prime },i^{\prime \prime }\in I,j^{\prime },j^{\prime \prime }\in J \end{array} \end{array}$$ (6)

$$\begin{array}{c} \sum_{k=1}^{m}\sum_{j=1}^{l}q_{ij}^{k}\geq N_{i},\forall i\in I \end{array}$$ (7)

$$\begin{array}{c} R_{j^{\prime },j^{\prime \prime }}^{i^{\prime },i^{\prime \prime }},q_{ij}^{k}\in \{0,1\},\forall i,j,k \end{array}$$ (8)

Constraint (4) means that each combat mission cannot be executed repeatedly during the battle process. Constraint (5) means that each battle cluster can only perform one combat task in each stage. Constraint (6) means that each combat cluster can not perform a combat task in a different combat stages. Constraint (7) indicates that the number of clusters that complete combat tasks can not be lower than the threshold of task completion. Constraint 8 is the decision variable constraint of the model, so the value of the variable can be between 0 and 1.

#### 3.2.3 Model

The model is given as follows:

$$\begin{array}{c} \left\{ \begin{array}{c} \min F_{1}\\ \max F_{2}\\ s.t.(4),(5),(6),(7),(8) \end{array}\right. \end{array}$$ (9)

For the MOCCTS model, the first objective function is multiplied by -1, i. e., from minimum optimization to maximum optimization. In the case of two maximized objective functions, in the objective space composed of the same decision variables, when one objective reaches the optimal value (maximum value), the other objective function can not reach the optimal value, which leads to the paradox that both objective functions taking the maximum value at the same time. Obviously, it is not appropriate to aggregate multiple objective functions into one objective by using the weight sum approach. From the search direction of the optimal value of the function, two objective functions form a two-dimensional objective space. In this space, a compromise solution is needed to find the solution set to deal with two objective functions simultaneously. The solution set is called Pareto front (PF) [45].

### 3.3 Definition and solution of cooperative time

In the combat process, there is the possibility of sudden changes in the environment, opponents and our fighting strength, which leads to certain fluctuations in the actual execution time of combat missions. If the execution time of task is fixed, it will be inconsistent with the actual situation, resulting in the solution result of the model not being operable. So, in this paper, the improved triangular fuzzy numbers (ITFN) is used to represent the execution time of combat mission, that is

$$\begin{array}{c} PT_{ij}^{k}=(pt_{ij}^{k}(1),pt_{ij}^{k}(2),pt_{ij}^{k}(3)) \end{array}$$ (10)

where $pt_{ij}^{k}(1),pt_{ij}^{k}(2),pt_{ij}^{k}(3)$ respectively represent the optimistic time, the most likely time, and the pessimistic time for performing combat tasks. If the membership function is used to represent $PT_{ij}^{k}$, that is

$$\begin{array}{c} \mu_{PT}\left(x\right)=\{\begin{array}{c} \frac{x-pt_{ij}^{k}(1)}{pt_{ij}^{k}(2)-pt_{ij}^{k}(1)},x\in [pt_{ij}^{k}(1),pt_{ij}^{k}(2))\\ \frac{x-pt_{ij}^{k}(3)}{pt_{ij}^{k}(2)-pt_{ij}^{k}(3)},x\in [pt_{ij}^{k}(2),pt_{ij}^{k}(3))\\ 0,others \end{array} \end{array}$$ (11)

In the previous studies on TFN, Sakawa et al. [46] proposed a measurement method and a linear operation method, and Seikh et al. [47] extended TFN in an intuitionistic fuzzy environment, and obtained the definition and calculation method of triangular intuitivistic fuzzy number (TIFN). On this basis, the operation mode of TFN is constantly being explored [48, 49]. In this paper, the structure of triangular fuzzy numbers is appropriately improved according to the needs of the model firstly, then a sort function of ITFN and a calculation method of sort probability are proposed.

**Fig 2:**
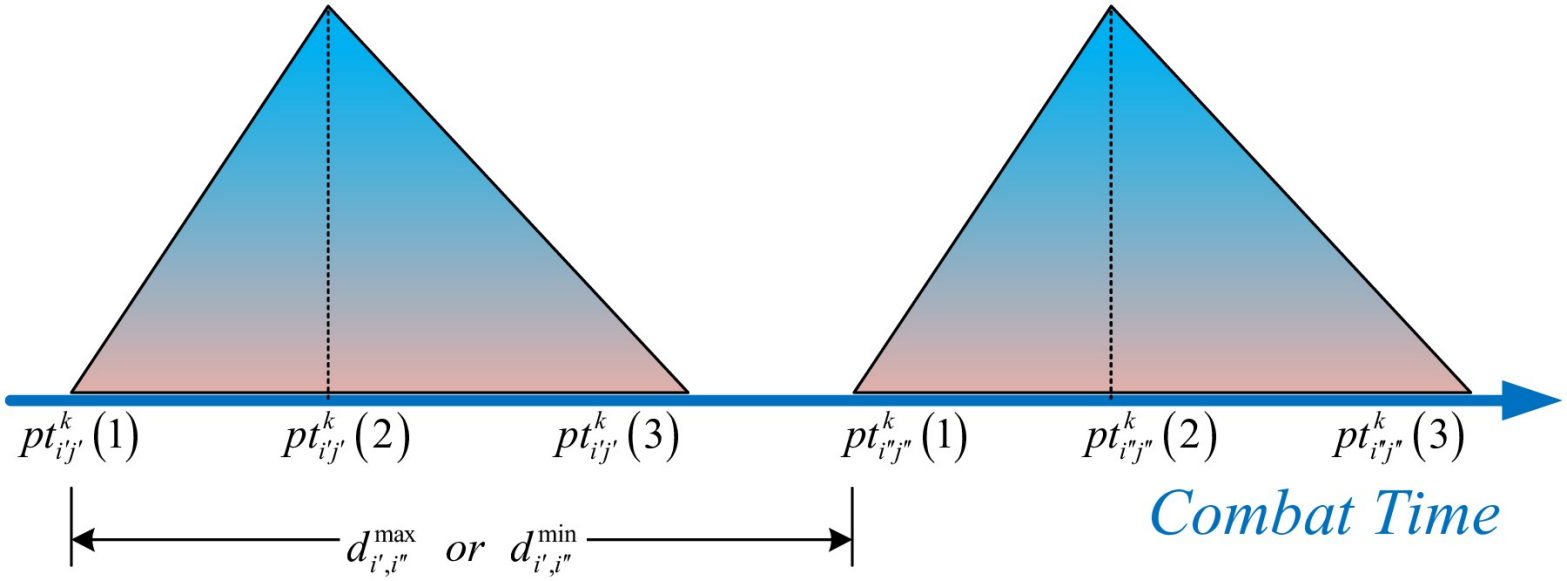
An illustration of using flexible time to improve TFN diagram.

**definition 1** *TFN is improved by using flexible time to define ITFN*. *Assuming that ITFN of task* *i*′ *and task* *i*″ *are* $PT_{i^{\prime }j^{\prime }}^{k}$ *and* $PT_{i^{\prime \prime }j^{\prime \prime }}^{k}$ *respectively, we define the maximum and minimum combat elastic times between the two tasks as* $d_{i^{\prime },i^{\prime \prime }}^{\max},d_{i^{\prime },i^{\prime \prime }}^{\min}$ *respectively, which means that if the maximum duration after the start of combat task* *i*′ *is* $d_{i^{\prime },i^{\prime \prime }}^{\max}$, *then combat task* *i*″ *must start*; *task* *i*″ *can only be started when the duration of task i is at least* $d_{i^{\prime },i^{\prime \prime }}^{\min}$. *As shown in* Fig 2.

$d_{i^{\prime },i^{\prime \prime }}=d_{i^{\prime },i^{\prime \prime }}^{\min}$ , if there is a minimum duration $d_{i^{\prime },i^{\prime \prime }}^{\min}$ between combat task *i*′ and task *i*″; If there is a longest duration $d_{i^{\prime },i^{\prime \prime }}^{\max}$ between combat task *i*′ and task *i*″, $d_{i^{\prime },i^{\prime \prime }}=d_{i^{\prime },i^{\prime \prime }}^{\max}$. Especially, if the combat task *i*′ and task *i*″ have multiple minimum durations $d_{i^{\prime },i^{\prime \prime }}^{\min}(1),d_{i^{\prime },i^{\prime \prime }}^{\min}(2),\cdots ,d_{i^{\prime },i^{\prime \prime }}^{\min}(N)$, then $d_{i^{\prime },i^{\prime \prime }}=\max\{d_{i^{\prime },i^{\prime \prime }}^{\min}(1),d_{i^{\prime },i^{\prime \prime }}^{\min}(2),\cdots ,d_{i^{\prime },i^{\prime \prime }}^{\min}(N)\}$; if there are multiple maximum durations $d_{i^{\prime },i^{\prime \prime }}^{\max}(1),d_{i^{\prime },i^{\prime \prime }}^{\max}(2),\cdots ,d_{i^{\prime },i^{\prime \prime }}^{\max}(N)$ between combat task *i*′ and task *i*″, $d_{i^{\prime },i^{\prime \prime }}=\max\{d_{i^{\prime },i^{\prime \prime }}^{\max}(1),d_{i^{\prime },i^{\prime \prime }}^{\max}(2),\cdots ,d_{i^{\prime },i^{\prime \prime }}^{\max}(N)\}$.

**definition 2** *The calculation rules of ITFN* [50]. *For the convenience of the following calculations, define four operations: equation, addition (subtraction), multiplication and division*.

*(1) Equation*. $PT_{i^{\prime }j^{\prime }}^{k^{\prime }}=PT_{i^{\prime \prime }j^{\prime \prime }}^{k^{\prime \prime }}$ *if and only if condition* $\left\{ \begin{array}{c} pt_{i^{\prime }j^{\prime }}^{k^{\prime }}(1)=pt_{i^{\prime \prime }j^{\prime \prime }}^{k^{\prime \prime }}(1)\\ pt_{i^{\prime }j^{\prime }}^{k^{\prime }}(2)=pt_{i^{\prime \prime }j^{\prime \prime }}^{k^{\prime \prime }}(2)\\ pt_{i^{\prime }j^{\prime }}^{k^{\prime }}(3)=pt_{i^{\prime \prime }j^{\prime \prime }}^{k^{\prime \prime }}(3) \end{array}\right.$ *is met*.
*(2) Addition (Subtraction)*. $PT_{i^{\prime }j^{\prime }}^{k^{\prime }}\pm PT_{i^{\prime \prime }j^{\prime \prime }}^{k^{\prime \prime }}=(pt_{i^{\prime }j^{\prime }}^{k^{\prime }}(1),pt_{i^{\prime }j^{\prime }}^{k^{\prime }}(2),pt_{i^{\prime }j^{\prime }}^{k^{\prime }}(3))\pm (pt_{i^{\prime \prime }j^{\prime \prime }}^{k^{\prime \prime }}(1),pt_{i^{\prime \prime }j^{\prime \prime }}^{k^{\prime \prime }}(2),pt_{i^{\prime \prime }j^{\prime \prime }}^{k^{\prime \prime }}(3))=(pt_{i^{\prime }j^{\prime }}^{k^{\prime }}(1)\pm pt_{i^{\prime \prime }j^{\prime \prime }}^{k^{\prime \prime }}(1),pt_{i^{\prime }j^{\prime }}^{k^{\prime }}(2)\pm pt_{i^{\prime \prime }j^{\prime \prime }}^{k^{\prime \prime }}(2),pt_{i^{\prime }j^{\prime }}^{k^{\prime }}(3)\pm pt_{i^{\prime \prime }j^{\prime \prime }}^{k^{\prime \prime }}(3))$ .
*(3) Multiplication*. $PT_{i^{\prime }j^{\prime }}^{k^{\prime }}\cdot PT_{i^{\prime \prime }j^{\prime \prime }}^{k^{\prime \prime }}=(pt_{i^{\prime }j^{\prime }}^{k^{\prime }}(1)\cdot pt_{i^{\prime \prime }j^{\prime \prime }}^{k^{\prime \prime }}(1),pt_{i^{\prime }j^{\prime }}^{k^{\prime }}(2)\cdot pt_{i^{\prime \prime }j^{\prime \prime }}^{k^{\prime \prime }}(2),pt_{i^{\prime }j^{\prime }}^{k^{\prime }}(3)\cdot pt_{i^{\prime \prime }j^{\prime \prime }}^{k^{\prime \prime }}(3))$ , *especially*, $\lambda PT_{i^{\prime }j^{\prime }}^{k^{\prime }}=\lambda (pt_{i^{\prime }j^{\prime }}^{k^{\prime }}(1),pt_{i^{\prime }j^{\prime }}^{k^{\prime }}(2),pt_{i^{\prime }j^{\prime }}^{k^{\prime }}(3))=(\lambda pt_{i^{\prime }j^{\prime }}^{k^{\prime }}(1),\lambda pt_{i^{\prime }j^{\prime }}^{k^{\prime }}(2),\lambda pt_{i^{\prime }j^{\prime }}^{k^{\prime }}(3))$.
*(4) Division*. $\frac{PT_{i^{\prime }j^{\prime }}^{k^{\prime }}}{PT_{i^{\prime \prime }j^{\prime \prime }}^{k^{\prime \prime }}}=\frac{\left(pt_{i^{\prime }j^{\prime }}^{k^{\prime }}(1),pt_{i^{\prime }j^{\prime }}^{k^{\prime }}(2),pt_{i^{\prime }j^{\prime }}^{k^{\prime }}(3)\right)}{\left(pt_{i^{\prime \prime }j^{\prime \prime }}^{k^{\prime \prime }}(1),pt_{i^{\prime \prime }j^{\prime \prime }}^{k^{\prime \prime }}(2),pt_{i^{\prime \prime }j^{\prime \prime }}^{k^{\prime \prime }}(3)\right)}=(\frac{pt_{i^{\prime }j^{\prime }}^{k^{\prime }}(1)}{pt_{i^{\prime \prime }j^{\prime \prime }}^{k^{\prime \prime }}(1)},\frac{pt_{i^{\prime }j^{\prime }}^{k^{\prime }}(2)}{pt_{i^{\prime \prime }j^{\prime \prime }}^{k^{\prime \prime }}(2)},\frac{pt_{i^{\prime }j^{\prime }}^{k^{\prime }}(3)}{pt_{i^{\prime \prime }j^{\prime \prime }}^{k^{\prime \prime }}(3)})$.

**definition 3** *Sorting function for ITFN*. *For* ∀*i* ∈ *I*, *j* ∈ *J*, *k* ∈ *K*, $PT_{ij}^{k}=(pt_{ij}^{k}(1),pt_{ij}^{k}(2),pt_{ij}^{k}(3))$, *the function* $f_{PT_{ij}^{k}}(x,y)=(pt_{ij}^{k}(3)-pt_{ij}^{k}(1))x+(pt_{ij}^{k}(2)-pt_{ij}^{k}(1))y+pt_{ij}^{k}(1),x,y\in [0,1]$ *calls the ITFN sorting function of* $PT_{ij}^{k}$.

**theorem 1** *Sorting probability degree of ITFN*. *For* ∀*i*′, *i*″ ∈ *I*, *j*′, *j*″ ∈ *J*, *k*′, *k*″ ∈ *K*, *S* *is the projection on the xoy surface of the three-dimensional Cartesion coordinates when the sorting function* $f_{PT_{i^{\prime }j^{\prime }}^{k^{\prime }}}(x,y)\geq f_{PT_{i^{\prime \prime }j^{\prime \prime }}^{k^{\prime \prime }}}(x,y)$. *S*′ *is the projection on the xoy surface of the three-dimensional Cartesion coordinates when the sorting function* $f_{PT_{i^{\prime }j^{\prime }}^{k^{\prime }}}(x,y)<f_{PT_{i^{\prime \prime }j^{\prime \prime }}^{k^{\prime \prime }}}(x,y)$, *the probability degree* $P(PT_{i^{\prime }j^{\prime }}^{k^{\prime }}\text{occursbefore}PT_{i^{\prime \prime }j^{\prime \prime }}^{k^{\prime \prime }})=\frac{S}{S+S^{\prime }}$, $P(PT_{i^{\prime \prime }j^{\prime \prime }}^{k^{\prime \prime }}\text{occursbefore}PT_{i^{\prime }j^{\prime }}^{k^{\prime }})=\frac{S^{\prime }}{S+S^{\prime }}$.

**Fig 3:**
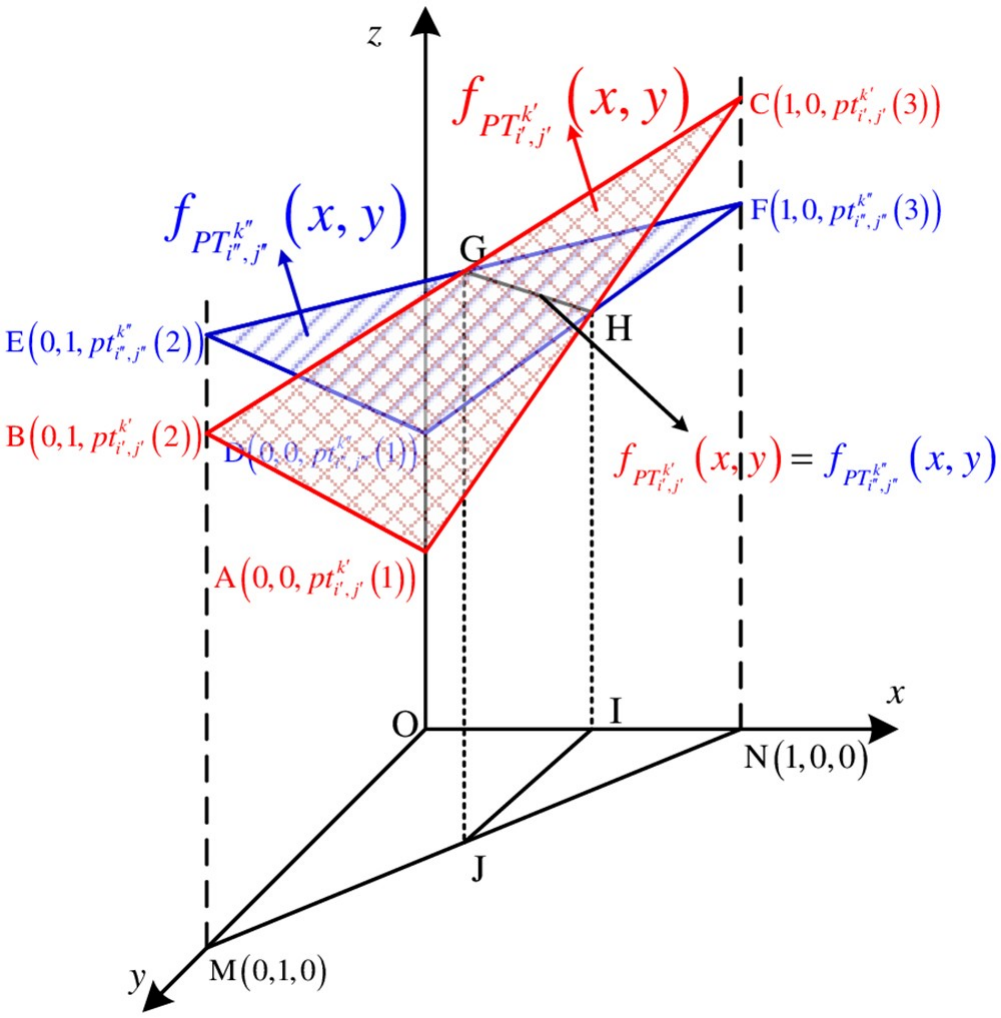
Schematic diagram for proving theorem 1.

**Proof 3.1 (Proof of Theorem 1)** *As shown in* Fig 3, *sorting functions* $f_{PT_{i^{\prime }j^{\prime }}^{k^{\prime }}}(x,y)$ and $f_{PT_{i^{\prime \prime }j^{\prime \prime }}^{k^{\prime \prime }}}(x,y)$ *are plotted in a three-dimensional cartesian coordinate system to obtain* ΔABC *and* ΔDEF. *The intersecting line of the two is* *GH*, *i.e*. $f_{PT_{i^{\prime }j^{\prime }}^{k^{\prime }}}(x,y)=f_{PT_{i^{\prime \prime }j^{\prime \prime }}^{k^{\prime \prime }}}(x,y)$. *As can be seen from* Fig 3, *S*_Δ*MON*_ = *S*_*ΔJIN*_ + *S*_*MOIJ*_ = *S* + *S*′, *so*, $P(PT_{i^{\prime }j^{\prime }}^{k^{\prime }}\text{occursbefore}PT_{i^{\prime \prime }j^{\prime \prime }}^{k^{\prime \prime }})=\frac{S_{\Delta JIN}}{S_{\Delta JIN}+S_{MOIJ}}=\frac{S}{S+S^{\prime }}$, $P(PT_{i^{\prime \prime }j^{\prime \prime }}^{k^{\prime \prime }}\text{occursbefore}PT_{i^{\prime }j^{\prime }}^{k^{\prime }})=\frac{S_{MOIJ}}{S_{\Delta JIN}+S_{MOIJ}}=\frac{S^{\prime }}{S+S^{\prime }}$.

**theorem 2** *Solution formula of probability degree*. *For* ∀*i*′, *i*″ ∈ *I*, *j*′, *j*″ ∈ *J*, *k*′, *k*″ ∈ *K*, *sorting functions* $f_{PT_{i^{\prime }j^{\prime }}^{k^{\prime }}}(x,y)=(pt_{i^{\prime }j^{\prime }}^{k^{\prime }}(3)-pt_{i^{\prime }j^{\prime }}^{k^{\prime }}(1))x+(pt_{i^{\prime }j^{\prime }}^{k^{\prime }}(2)-pt_{i^{\prime }j^{\prime }}^{k^{\prime }}(1))y+pt_{i^{\prime }j^{\prime }}^{k^{\prime }}(1)$ *and* $f_{PT_{i^{\prime \prime }j^{\prime \prime }}^{k^{\prime \prime }}}(x,y)=(pt_{i^{\prime \prime }j^{\prime \prime }}^{k^{\prime \prime }}(3)-pt_{i^{\prime \prime }j^{\prime \prime }}^{k^{\prime \prime }}(1))x+(pt_{i^{\prime \prime }j^{\prime \prime }}^{k^{\prime \prime }}(2)-pt_{i^{\prime \prime }j^{\prime \prime }}^{k^{\prime \prime }}(1))y+pt_{i^{\prime \prime }j^{\prime \prime }}^{k^{\prime \prime }}(1)$, *x*, *y* ∈ [0, 1], *let* $A=pt_{i^{\prime }j^{\prime }}^{k^{\prime }}(1),B=pt_{i^{\prime }j^{\prime }}^{k^{\prime }}(2),C=pt_{i^{\prime }j^{\prime }}^{k^{\prime }}(3),D=pt_{i^{\prime \prime }j^{\prime \prime }}^{k^{\prime \prime }}(1),E=pt_{i^{\prime \prime }j^{\prime \prime }}^{k^{\prime \prime }}(2),F=pt_{i^{\prime \prime }j^{\prime \prime }}^{k^{\prime \prime }}(3)$, *then, the formulae for solving the ITFN probability degree under different conditions is shown in* Table 2.

**Table 2:** Possibility solving formulae for different ITFN.

The size relationship of $PT_{i^{\prime }j^{\prime }}^{k^{\prime }}$ and $PT_{i^{\prime \prime }j^{\prime \prime }}^{k^{\prime \prime }}$	Solution result of probability
$\left\{ \begin{array}{c} pt_{i^{\prime }j^{\prime }}^{k^{\prime }}(1)=pt_{i^{\prime \prime }j^{\prime \prime }}^{k^{\prime \prime }}(1)\\ pt_{i^{\prime }j^{\prime }}^{k^{\prime }}(2)\geq pt_{i^{\prime \prime }j^{\prime \prime }}^{k^{\prime \prime }}(2)\\ pt_{i^{\prime }j^{\prime }}^{k^{\prime }}(3)\geq pt_{i^{\prime \prime }j^{\prime \prime }}^{k^{\prime \prime }}(3) \end{array}\right.$	$P(PT_{i^{\prime }j^{\prime }}^{k^{\prime }}\text{occursbefore}PT_{i^{\prime \prime }j^{\prime \prime }}^{k^{\prime \prime }})=1$
$\left\{ \begin{array}{c} pt_{i^{\prime }j^{\prime }}^{k^{\prime }}(1)\leq pt_{i^{\prime \prime }j^{\prime \prime }}^{k^{\prime \prime }}(1)\\ pt_{i^{\prime }j^{\prime }}^{k^{\prime }}(2)\leq pt_{i^{\prime \prime }j^{\prime \prime }}^{k^{\prime \prime }}(2)\\ pt_{i^{\prime }j^{\prime }}^{k^{\prime }}(3)\leq pt_{i^{\prime \prime }j^{\prime \prime }}^{k^{\prime \prime }}(3) \end{array}\right.$	$P(PT_{i^{\prime \prime }j^{\prime \prime }}^{k^{\prime \prime }}\text{occursbefore}PT_{i^{\prime }j^{\prime }}^{k^{\prime }})=1$
$\left\{ \begin{array}{c} pt_{i^{\prime }j^{\prime }}^{k^{\prime }}(1)\leq pt_{i^{\prime \prime }j^{\prime \prime }}^{k^{\prime \prime }}(1)\\ pt_{i^{\prime }j^{\prime }}^{k^{\prime }}(2)\leq pt_{i^{\prime \prime }j^{\prime \prime }}^{k^{\prime \prime }}(2)\\ pt_{i^{\prime }j^{\prime }}^{k^{\prime }}(3)>pt_{i^{\prime \prime }j^{\prime \prime }}^{k^{\prime \prime }}(3) \end{array}\right.$	$\left\{ \begin{array}{c} P(PT_{i^{\prime }j^{\prime }}^{k^{\prime }}\text{occursbefore}PT_{i^{\prime \prime }j^{\prime \prime }}^{k^{\prime \prime }})=\frac{{\left(C-F\right)}^{2}}{\left(C+E-B-F)(C+D-A-F\right)}\\ P(PT_{i^{\prime \prime }j^{\prime \prime }}^{k^{\prime \prime }}\text{occursbefore}PT_{i^{\prime }j^{\prime }}^{k^{\prime }})=\frac{(C+E-B-F)(C+D-A-F)-{\left(C-F\right)}^{2}}{\left(C+E-B-F)(C+D-A-F\right)} \end{array}\right.$
$\left\{ \begin{array}{c} pt_{i^{\prime }j^{\prime }}^{k^{\prime }}(1)<pt_{i^{\prime \prime }j^{\prime \prime }}^{k^{\prime \prime }}(1)\\ pt_{i^{\prime }j^{\prime }}^{k^{\prime }}(2)>pt_{i^{\prime \prime }j^{\prime \prime }}^{k^{\prime \prime }}(2)\\ pt_{i^{\prime }j^{\prime }}^{k^{\prime }}(3)\leq pt_{i^{\prime \prime }j^{\prime \prime }}^{k^{\prime \prime }}(3) \end{array}\right.$	$\left\{ \begin{array}{c} P(PT_{i^{\prime }j^{\prime }}^{k^{\prime }}\text{occursbefore}PT_{i^{\prime \prime }j^{\prime \prime }}^{k^{\prime \prime }})=\frac{{\left(B-E\right)}^{2}}{\left(D+B-E-A)(F+B-E-C\right)}\\ P(PT_{i^{\prime \prime }j^{\prime \prime }}^{k^{\prime \prime }}\text{occursbefore}PT_{i^{\prime }j^{\prime }}^{k^{\prime }})=\frac{(D+B-E-A)(F+B-E-C)-{\left(B-E\right)}^{2}}{\left(D+B-E-A)(F+B-E-C\right)} \end{array}\right.$
$\left\{ \begin{array}{c} pt_{i^{\prime }j^{\prime }}^{k^{\prime }}(1)<pt_{i^{\prime \prime }j^{\prime \prime }}^{k^{\prime \prime }}(1)\\ pt_{i^{\prime }j^{\prime }}^{k^{\prime }}(2)>pt_{i^{\prime \prime }j^{\prime \prime }}^{k^{\prime \prime }}(2)\\ pt_{i^{\prime }j^{\prime }}^{k^{\prime }}(3)>pt_{i^{\prime \prime }j^{\prime \prime }}^{k^{\prime \prime }}(3) \end{array}\right.$	$\left\{ \begin{array}{c} P(PT_{i^{\prime }j^{\prime }}^{k^{\prime }}\text{occursbefore}PT_{i^{\prime \prime }j^{\prime \prime }}^{k^{\prime \prime }})=\frac{{\left(D-A\right)}^{2}}{\left(C+D-A-F)(B+D-A-E\right)}\\ P(PT_{i^{\prime \prime }j^{\prime \prime }}^{k^{\prime \prime }}\text{occursbefore}PT_{i^{\prime }j^{\prime }}^{k^{\prime }})=\frac{(C+D-A-F)(B+D-A-E)-{\left(D-A\right)}^{2}}{\left(C+D-A-F)(B+D-A-E\right)} \end{array}\right.$

**Proof 3.2 (Proof of Theorem 2)** *The following proof illustrates only the third case in* Table 2. *As shown in* Fig 3, *the end point of the projection* *JI* *of the intersecting line GH is on the line MN, and the coordinate of the end point is* (*x*_0_, *y*_0_), *then the coordinate should be the solution of the following equation*.

$$\begin{array}{c} \left\{ \begin{array}{c} A+(B-A)y_{0}+(C-A)x_{0}=D+(E-D)y_{0}+(F-D)x_{0}\\ y_{0}+x_{0}=1 \end{array}\right. \end{array}$$ (12)

*The endpoint coordinate is* $\left(\frac{B-E}{F+B-E-C},\frac{F-C}{F+B-E-C}\right)$. *Similarly, it can be solved when the endpoint of the projection JI is on the line segments OM and ON, the endpoint coordinates are* $\left(0,\frac{D-A}{B+D-A-E}\right)$, $\left(\frac{D-A}{C+D-A-F},0\right)$. *The formula for solving the possibility degree can be obtained by substitution*.

## 4 A multi-objective improved bat algorithm based on angle decomposition

As shown above, the MOOCTS model is in fact a multi-objective optimization problem (MOP). In the literature, evolutionary multi-objective optimization algorithms (EMOAs), such as NSGA-II and MOEA/D, are reportedly effective to solve problems with two or three objective functions, but poor preformance are observed when they are used to address MaOPs. Algorithmic improvements to EMOAs are therefore needed for MaOPs. To date, the algorithmic improvements are usually made from three aspects: (1) Convergence acceleration: usually new dominance theory or modified Pareto dominance relationship is developed in this type of algorithms. See, for example, [51–53], can be significantly enhanced by different convergence enhancement mechanisms. (2) Decomposition: This type of algorithms works by decomposing the multi-objective problem into several single-objective problems. Typical examples of such algorithms include weight setting [54], decomposition mechanism [55, 56], generation of offspring and environment selection [57]. (3) Evaluation criteria: This type of algorithms improves the convergence and diversity of Pareto solutions by developing appropriate performance criteria [58].

In this section, inspired by the MOEA/D mechanism, we improved the decomposition mechanism of the algorithm and the population updating strategy, and proposed the MOIBA/AD algorithm.

### 4.1 Main motivation and innovation

(1) In the literature, MOEA/D is one of the most widely used algorithm for solving MaOPs. The main idea is to combine multiple objectives into one single objective and solve this proxy objective to obtain the Pareto front. The three commonly used aggregation methods are weight sum approach, Tchebycheff approach [59] and penalty-based boundary intersection approach [60]. One major limitation of the three methods is that the time complexity of problem solving increases linearly (for the former method) or non-linearly (for the latter two methods) with the number of objective functions, thereby limiting the size of problems that can be solved.
(2) For MOPs, the widely used methods for individual selection include entropy-based methods [61], grid-based methods [62] and cluster-based methods [63], amont others. Although these methods can produce desirable distributions of population on some specific multi-objective problems, they generally do not produce an uniform distribution of Pareto front on a general MaOP.
(3) In almost all existing MOEA/D algorithms, the simulated binary crossover operator (SBX) is used for population update. However, this method has two limitations: 1) SBX reduces the population diversity. In the early stage of the search, the search space is usually small. For multi-objective problems with complex Pareto sets, SBX would probably eliminate the diversity of the population. 2) SBX often leads to inferior solutions to MOEAs.

Our proposed algorithm differs from existing algorithms in three aspects.

- Firstly, we decompose the target space based on angle decomposition. Based on uniformly distributed unit vectors, the target space is divided into multiple sub-regions, and only the non-dominated individuals having the smallest angle with the weight vector will be retained. Through this update strategy, the Pareto front is decomposed into several fronts, and the PF of each subregion is linearly approximated without any aggregation function. This is different from the aggregation strategy of multiple strategies in traditional MOEA/D and helps speed up the calculation.
- Secondly, we developed a strategy to improve the distribution of the population. We combine the individual dominance relationship with our proposed population selection and replacement strategy to obtain offspring individuals with greater diversity.
- Lastly, we improve the traditional MOEA/D by taking advantage of the IBA, which combines local search with global search. Moreover, we apply random perturbations to the speed and position updating in BA, so as to prevent the search from getting trapped in local optima.

To sum up, the MOIBA/D continues the framework of the traditional MOEA/D algorithm, that is, the solution space is decomposed by appropriate decomposition strategy, and the required PF solution set is finally obtained by constantly updating the initial population (selection and replacement strategies) in the solution space. On the basis of this framework, in order to reduce the time complexity of the algorithm, we adopt the strategy of angle decomposition to the solution space, and on this basis, we put forward the strategy of population selection and updating of solution space based on angle decomposition. The IBA proposed in this paper is used in the specific implementation of the population individual optimization process.

### 4.2 Introduction of MOIBA/AD strategy

#### 4.2.1 Method of target space decomposition and population classification

Given a set of uniformly distributed direction vectors **λ**^1^, **λ**^2^, ⋯, **λ**^*N*^, optimization target dimension is *M*, the objective space Y of the multi-objective optimization problem can be decomposed into $Y_{1},Y_{2},\cdots ,Y_{N}$ according to formula (13) and (14), and the population *Pop* is divided into $P^{1},P^{2},\cdots ,P^{N}$ by Eq (15).

$$\begin{array}{c} \begin{array}{cc} Y_{i}=\{F(x)|x\in Pop,\cos(F(x)-Z,\lambda^{f}) & >\underset{f\neq g}{\cos}(F(x)-Z,\lambda^{g})\},\\ & f=1,2,\cdots ,N,g=1,2,\cdots ,M \end{array} \end{array}$$ (13)

$$\begin{array}{c} \cos(F(x)-Z,\lambda^{f})=\frac{\lambda^{f}\cdot {\left(F(x)-Z\right)}^{T}}{\left|\lambda^{f}||F(x)-Z\right|},\forall f \end{array}$$ (14)

$$\begin{array}{c} \begin{array}{c} P^{f}=\{x|x\in Pop,\cos(F(x)-Z,\lambda_{f})>\cos_{f\neq g}(F(x)-Z,\lambda_{g})\},\forall f,g \end{array} \end{array}$$ (15)

where *F*(*x*) represent fitness functions, ***Z*** = (*Z*_1_, *Z*_2_, ⋯, *Z*_*M*_) represent a reference point, *Z*_*j*_ = min{*f*_*j*_(*x*)|*x*∈*Pop*}, *f*_*j*_(*x*) is the *j*^*th*^ optimization objective function.

**Fig 4:**
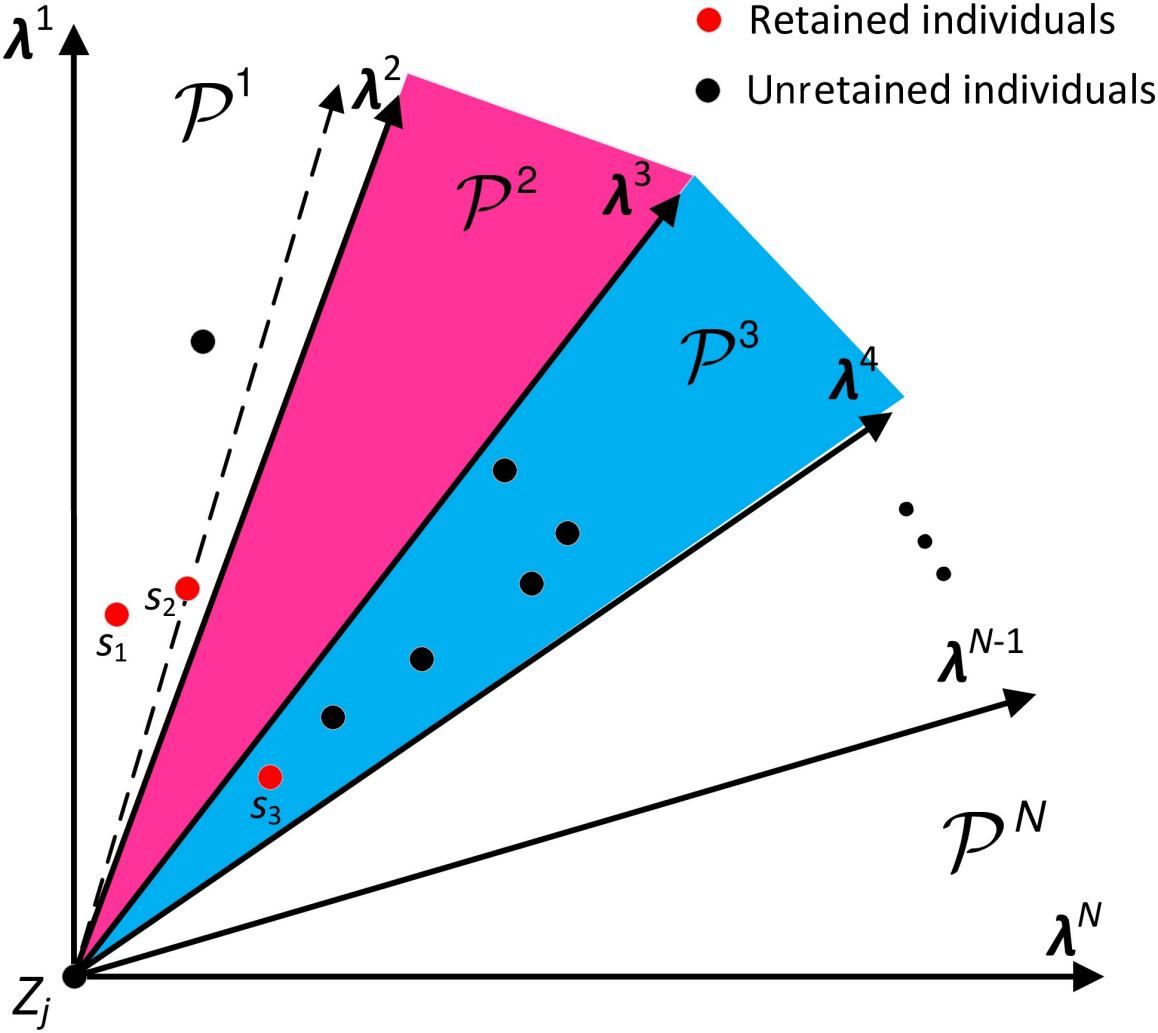
An illustration of the 2-dimensional target space decomposition.

In this way, the target space was divided by angle decomposition and the populations were classified, which was ready for the next update and screening. Taking the two-dimensional target space as an example, the spatial decomposition diagram was shown in Fig 4.

#### 4.2.2 Population selection and replacement strategy

$P^{f}\left(f=1,2,\cdots ,N\right)$ is updated after the population classification is completed, in order to keep only one individual in each $P^{f}$ that is well divided.

If the sub-region $Y_{f}$ contains an individual in $P^{f}$, (1) if only one individual is contained, the individual is retained; (2) if there were more than one individual, all the individuals were subjected to non-dominated sorting [64]. The non-dominated individual was preferably selected, and then the individual with the smallest angle with the non-dominated individual was selected according to Eq (16).

$$\begin{array}{c} \begin{array}{c} P^{f}=\{x|x\in P^{f},\cos(F(x)-Z,\lambda_{f})>\cos_{f\neq g}(F(x)-Z,\lambda_{j})\},\forall f,g \end{array} \end{array}$$ (16)

If the sub-region $Y_{f}$ did not contain the individuals in $P^{f}$, the individual corresponding to the target vector with the smallest angle with **λ**^*i*^ was selected from the current population and retained as the representative individual in $P^{f}$, while the individuals in the remaining region $Y_{f}$ were discarded. The population update diagram is shown in Fig 4. The solution space is decomposed into N regions by N direction vectors, and the individuals in the solution space is also classified into N parts. In the subspaces $P^{1},P^{2},P^{3}$, there are multiple individuals in $P^{1}$ and $P^{3}$, but no individual in $P^{2}$. In this case, $P^{1}$ and $P^{3}$ make individual selection through non-dominated sorting, and the individuals are reserved as *s*_1_ and *s*_2_ marked in red. In $P^{2}$, the individual corresponding to the target vector with the smallest angle of **λ**^2^ is reserved, and the reserved individual is the *s*_2_ marked in red in $P^{1}$.

#### 4.2.3 Optimization method of IBA

The traditional bat algorithm is inspired by the echolocation principle of bats looking for targets [65]. It controls the bat position and flight speed by controlling the frequency, loudness and rate of the transmitted pulses of the bats, which well combines the global optimization with the local optimization. In order to further improve the optimization ability of the algorithm and maintain the population distribution, the following improvements are made on the basis of the traditional bat algorithm.

In BA, the frequency adjustment formula for bats is:

$$\begin{array}{c} f_{f}=f_{min}+\left(f_{max}-f_{min}\right)\beta \end{array}$$ (17)

where *f* = 1, 2, ⋯, *N*, represents an individual of a bat population.

(1) Improvement of global optimization for bats.
  The improved speed update formula is:
  $$\begin{array}{c} v_{f}^{t+1}=\{\begin{array}{c} \omega v_{f}^{t}+f_{f}(x_{f}^{t}-v_{f}^{*}),x_{f}^{t}≻{\bar{x}}^{t},\forall f\\ v_{f}^{t}+f_{f}(x_{f}^{t}-v_{f}^{*}),x_{f}^{t}≺{\bar{x}}^{t},\forall f \end{array} \end{array}$$ (18)
  In the iterative process of the algorithm, whether the position of the current individual is better than the average value is judged according to the average value ${\bar{x}}^{t}$ of all individual positions in the current bat population; if the position of the current individual is better than the average value, the updating speed of the first formula in formula (18) is adopted. Otherwise, the updating speed of the second formula is adopted. In the first formula, the flying speed of the bat is added with a disturbance coefficient *ω*, *ω* = *ω*_max_ − (*ω*_max_ − *ω*_min_) · *iter*/*iter*_max_, where *ω*_max_, *ω*_min_ represent the maximum and minimum values of the disturbance coefficient, and *iter*, *iter*_max_ represent the current number of iterations and the maximum number of iterations of the bat population. The velocity variable of the previous generation is multiplied by the velocity coefficient *ω* to disturb the individual velocity, so as to prevent it from flying to the local optimum and avoid the algorithm from falling into the local optimum. When the individual position of the bat is worse than the population average, the original formula is still used to update the speed to accelerate the convergence of the algorithm, which can help the individual in the disadvantaged position to fly to the optimal position quickly.
  The improved location update formula is:
  $$\begin{array}{c} x_{f}^{t+1}=x_{f}^{t}+[A_{m}\cos(2\pi f_{f}t)]\cdot v_{f}^{t+1},\forall f \end{array}$$ (19)
  Compare with the traditional position update formula of BA algorithm, the periodic function is introduced to replace the fixed coefficient value, which is the amplitude of the trigonometric function, to spread the fluctuations of the bat’s acoustic frequency to the changes of the bat’s position, so that the bat’s position maintains the ability of continuous update, and thus maintains the diversity and distribution of the population.
(2) Improvement of local optimization for bats.
  $$\begin{array}{c} X^{t+1}=X^{t}+{\bar{A}}^{t}⊕\varepsilon \end{array}$$ (20)
  In the local optimization method, the random Gaussian disturbance is added, where *ε* is of the same order as the current population matrix ***X***^*t*^, *ε*_*i*_ ∼ *N*(0, 1). To avoid excessive fluctuation, ${\bar{A}}^{t}$ represents the average loudness of all bat individuals currently emitted, and ${\bar{A}}^{t}$ is used to adjust the search range of *ε*_*f*_.
  Local optimization and global optimization are linked by pulse transmit loudness and rate:
  $$\begin{array}{c} A_{f}^{t+1}=\alpha A_{f}^{t},\forall f \end{array}$$ (21)
  $$\begin{array}{c} r_{f}^{t+1}=r_{f}^{0}[1-\exp(-\gamma t)],\forall f \end{array}$$ (22)
  where *α*, *γ* represent that rate of change of loudness and the rate of change of pulse emission, both of which are constant. Obviously, ∀0 < *α* < 1, *γ* > 0, *t* → ∞, $A_{f}^{t}\to 0$, $r_{f}^{t}\to r_{f}^{0}$.

#### 4.2.4 Population selection strategy

For individual selection of the updated population of each generation, in addition to the updating of the individual position and speed according to the updating strategy in Section 4.2.3, it is necessary to select and trim individuals according to the population size in the updated population.

**Fig 5:**
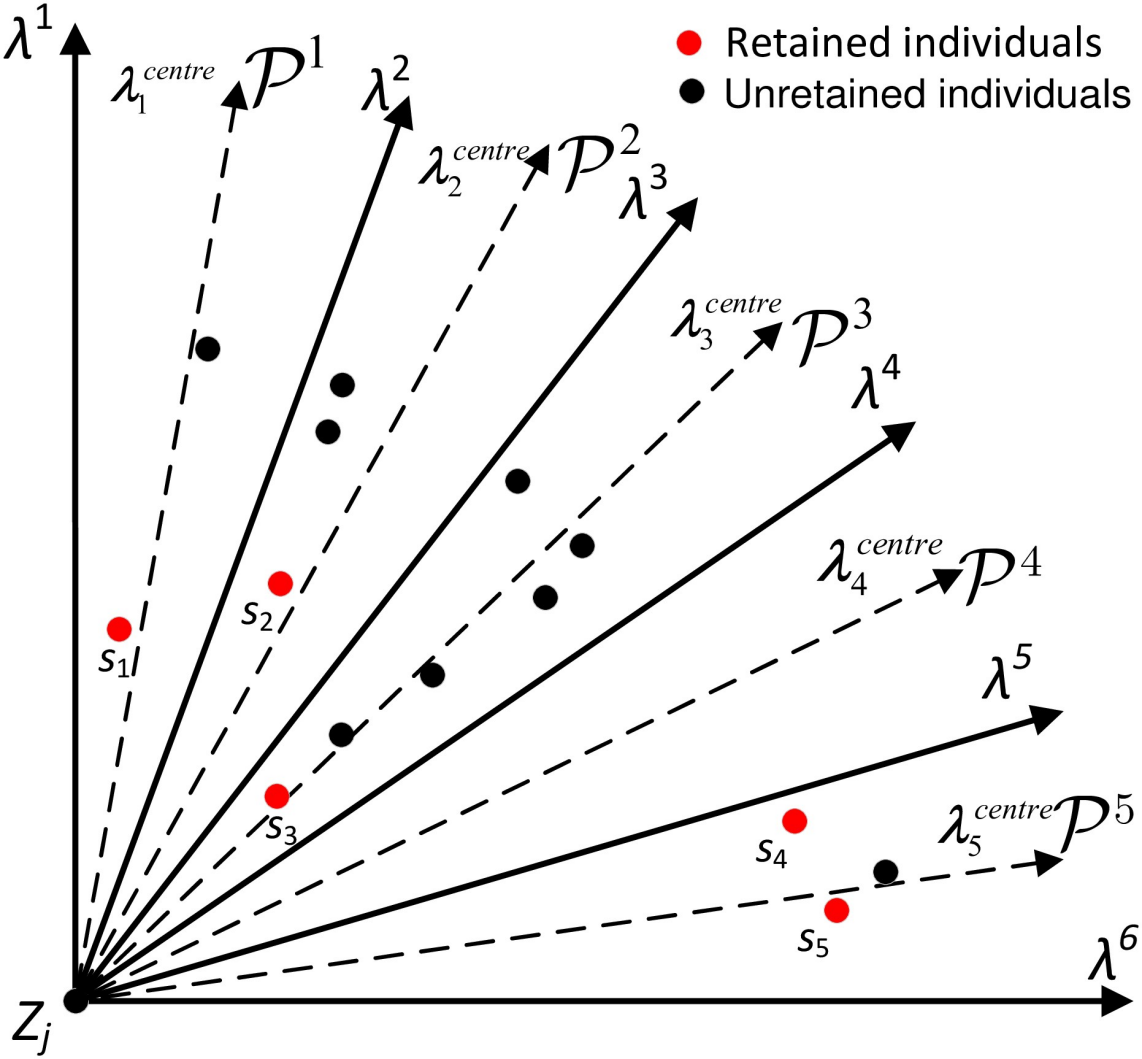
A illustration of the individual selection.

In order to better explore the sparse region, the individual congestion generated by the local optimization method according to Eq (19) is sorted, and then the individual selection is conducted. In the global optimization process, the initial selection is firstly performed according to Eqs (16)–(19). Next, for the preservation of population distribution, the following selection method based on unit neighborhood vector is proposed:

Let $\lambda^{centre}=(\lambda_{1}^{centre},\lambda_{2}^{centre},\cdots ,\lambda_{M}^{centre})$, where $\lambda_{g}^{centre}$ is calculated as:

$$\begin{array}{c} \lambda_{g}^{centre}=\frac{1}{T}(\lambda_{g}^{f_{1}}+\lambda_{g}^{f_{2}}+\cdots +\lambda_{g}^{f_{T}}) \end{array}$$ (23)

where $\lambda_{g}^{centre}\left(g=1,2,\cdots ,M\right)$ is the *g*-dimensional component of **λ**^*centre*^, and $\lambda^{f_{1}},\lambda^{f_{2}},\cdots ,\lambda^{f_{T}}$ (*f* = 1, 2, ⋯, *N*) is the *T* unit neighborhood vectors of unit vector **λ**^*f*^. In this way, the individual with the smallest angle with **λ**^*centre*^ is selected by Eq (23), which greatly improves the population distribution. A five-dimensional target was taken as an example, and the selection diagram of the population was shown in Fig 5. We first select *s*_1_, *s*_2_, *s*_3_ and *s*_5_ for the regions $P^{1}$, $P^{2}$, $P^{3}$ and $P^{5}$ according to (16)–(19). In region $P^{4}$, there is no candidate individuals available for selection. In this case, we used the proposed method based on unit neighborhood vector to select *s*_4_ as the retained individual for regions $P^{1}$.

### 4.3 MOIBA/AD framework for MOOCTS

#### 4.3.1 Population coding scheme and spatial transformation strategy for solving MOOCTS

In the introduction, many papers use binary code and Gray code when studying this kind of problem, but the algorithm of solving model in this paper is completely combined with BA algorithm, and its population search and optimization operation belong to the category of real number operation, so this paper adopts real number code. Since the sequence number set of the combat task is *I* = {1, 2, ⋯, *n*} and the sequence number set of the combat cluster is *K* = {1, 2, ⋯, *m*}, the code vectors are *X*_*I*_ = (*x*_*I*1_, *x*_*I*2_, ⋯, *x*_*In*_), *X*_*K*_ = (*x*_*K*1_, *x*_*K*2_, ⋯, *x*_*Km*_) respectively.

Regarding population initialization in Algorithm 1, we propose a strategy to transform discrete space into continuous space, rather than apply random initialization, which is the most commonly used method for creating the initial population. The basic idea of our strategy is to transform the binary values in discrete space into some intermediate variables, which will then be converted into real values in continuous space. At the heart of our strategy is determining an appropriate function for spatial transformation. Our strategy consists of three steps, which are shown below.

**Step1**: After population initialization, we choose the sigmoid function as the function for spatial transformation and calculate a value for each individual by applying this function. The sigmoid function is given by
  $$\begin{array}{c} sig(x)=\frac{1}{1+e^{-x}} \end{array}$$ (24)
**Step2**: Save all populations in the intermediate variable *Pop*_*mid*_.
**Step3**: Transform the intermediate variables into binary values according to $x_{f}=\{\begin{array}{c} 1,sig(x_{f})>0.5\\ 0,otherwise \end{array},f=1,2,\cdots ,N$.

With this procedure, the continuous space is transformed into discrete space.

#### 4.3.2 Algorithm step

**Fig 6:**
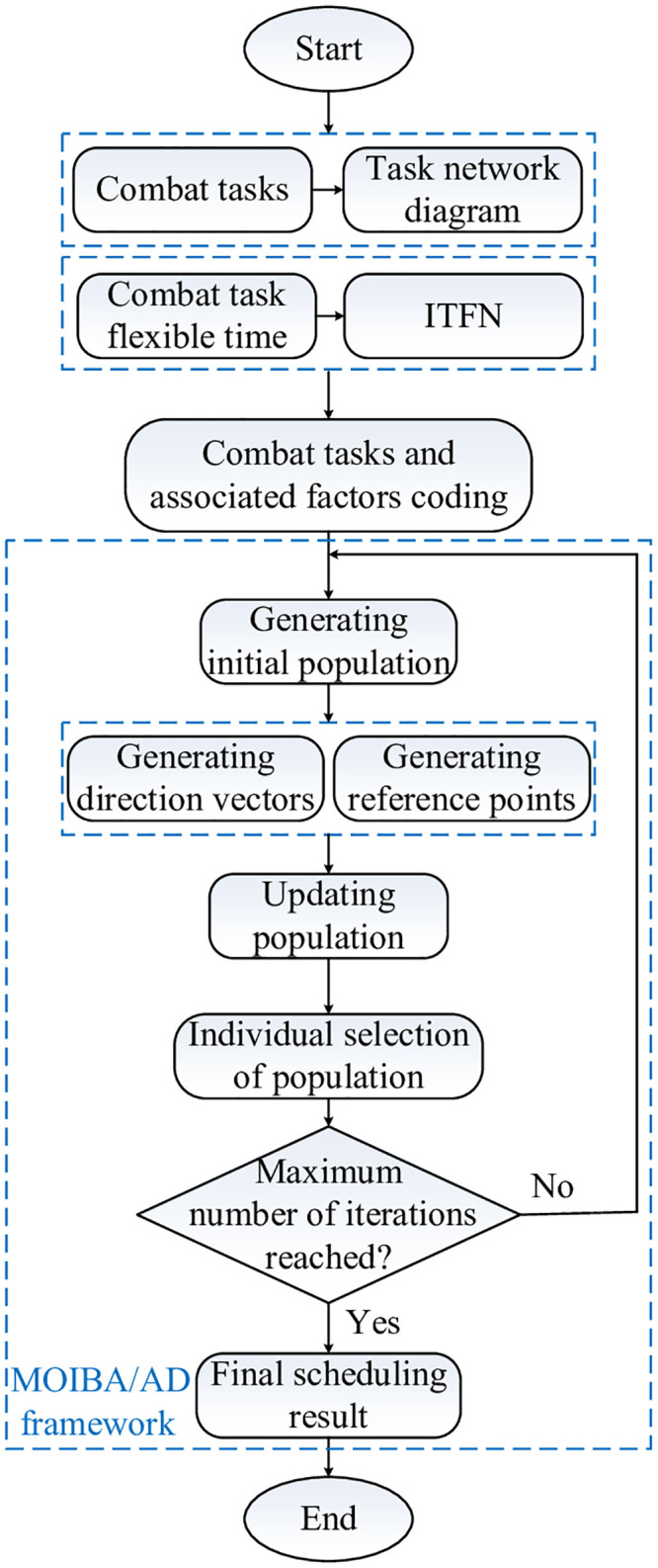
A illustration of the whole MOOCTS model solving process.

Under the above strategy, the steps of MOIBA/AD algorithm proposed in this paper are as **Algorithm 1**. In the framework of MOIBA/AD algorithm, the operational task scheduling model, ITFN and other factors are added into the process of problem solving. The whole MOOCTS model solving process is shown in Fig 6.

#### 4.3.3 Computational complexity of MOIBA/AD

In order to further illustrate the efficiency of the algorithm, the analysis of MOIBA/AD step shows that its time complexity depends on the construction and generation method of Pareto optimal solution set, which mainly comes from the updating of individuals and reference points in Step2. In Step1.4, the euclidean distance between direction vectors λ^1^, λ^2^, ⋯, λ^*N*^ is calculated, the complexity is *O*(*N*^2^), and the complexity in the initialization process of Step1 is *kO*(*N* ⋅ *M*) + *O*(*N*^2^). In the process of population updating, the complexity of updating individuals and reference points is *O*(*D* ⋅ *N* ⋅ *M* ⋅ *T*) by using the improved BA, where *D* is the dimension in IBA, *T* is the number of unit neighbor vectors, as *T* ≤ *N*, *M* ≤ *N*, the time complexity of MOIBA/AD is *O*(*D* ⋅ *N* ⋅ *M* ⋅ *T*), which indicates that MOIBA/AD is computationally efficient.

**Algorithm 1 MOIBA/AD**

**Input**: MOP, set of direction vectors {λ^1^, λ^2^, ⋯, λ^*N*^}, the reference point *Z*, the maximum number of iterations *iter*_max_, initial loudness of pulse *A*^0^, initial pulse emission rate *r*^0^, change of pulse loudness rate *α*, change of pulse transmission frequency *γ*, initial pulse frequency *f*_0_, maximum pulse frequency *f*_*max*_, minimum pulse frequency *f*_*min*_, individual initial velocity *v*_0_, maximum and minimum values of disturbance coefficient of individual velocity *ω*_max_,*ω*_min_, amplitude of position disturbance function *A*_*m*_.

**Output**: optimal *EP*;

1: **Step 1** initializing;

2: Step1.1 set an external noninferior solution *EP* = ∅;

3: Step1.2 generating *N* uniformly distributed direction vectors **λ**^1^, **λ**^2^, ⋯, **λ**^*N*^, according to the population size *N*, number of iterations *iter* = 0;

4: Step1.3 generate initial population *Pop*(0), initial speed *v*_0_;

5: Step1.4 calculating the euclidean distance between **λ**^*i*^ and **λ**^*j*^, *i*, *j* = 1, 2, ⋯, *N*, determining *T* unit neighbor vectors $\lambda^{i_{1}},\lambda^{i_{2}},\cdots ,\lambda^{i_{T}}$;

6: Step1.5 determine an initial reference point $Z^{0}=(Z_{1}^{0},Z_{2}^{0},\cdots ,Z_{M}^{0})$, where $Z_{j}^{0}=\text{min}\{f_{j}(x)|x\in Pop(0)\}$, *j* = 1, 2, ⋯, *M*;

7: **Step2** population update;

8: Step2.1 update the individuals in *Pop*(*iter*) according to the update strategy of 3.2.2,so that only one individual is reserved in each divided *P*^*i*^, and finally *N* individuals are selected;

9: Step2.2 updating the progeny population according to the formula (17)–(20), storing the updated *N* individuals in *EP*,*Pop*(*iter*) = *Pop*(*iter*) ∪ *EP*, and updating the reference point;

10: Step2.3 select *N* individuals from *Pop*(*iter*) as *Pop*(*iter*+ 1) according to the selection strategy in 3.2.4, *iter* = *iter* + 1, and update *EP*;

11: **Step3** jud whether that *iter* = *iter*_max_ is satisfy, if so, terminating the algorithm and outputting *EP*; if not, return to Step2.

## 5 Experiment and result analysis

The following is a practical example to solve the problem of cooperative time scheduling in land offensive operations. The algorithm proposed in this paper with some compared algorithms are used for simulation verification and result analysis. The simulation environment is CPU i7–8850H, 16.0GB RAM, operating system Windows10.

### 5.1 Simulation example

**Fig 7:**
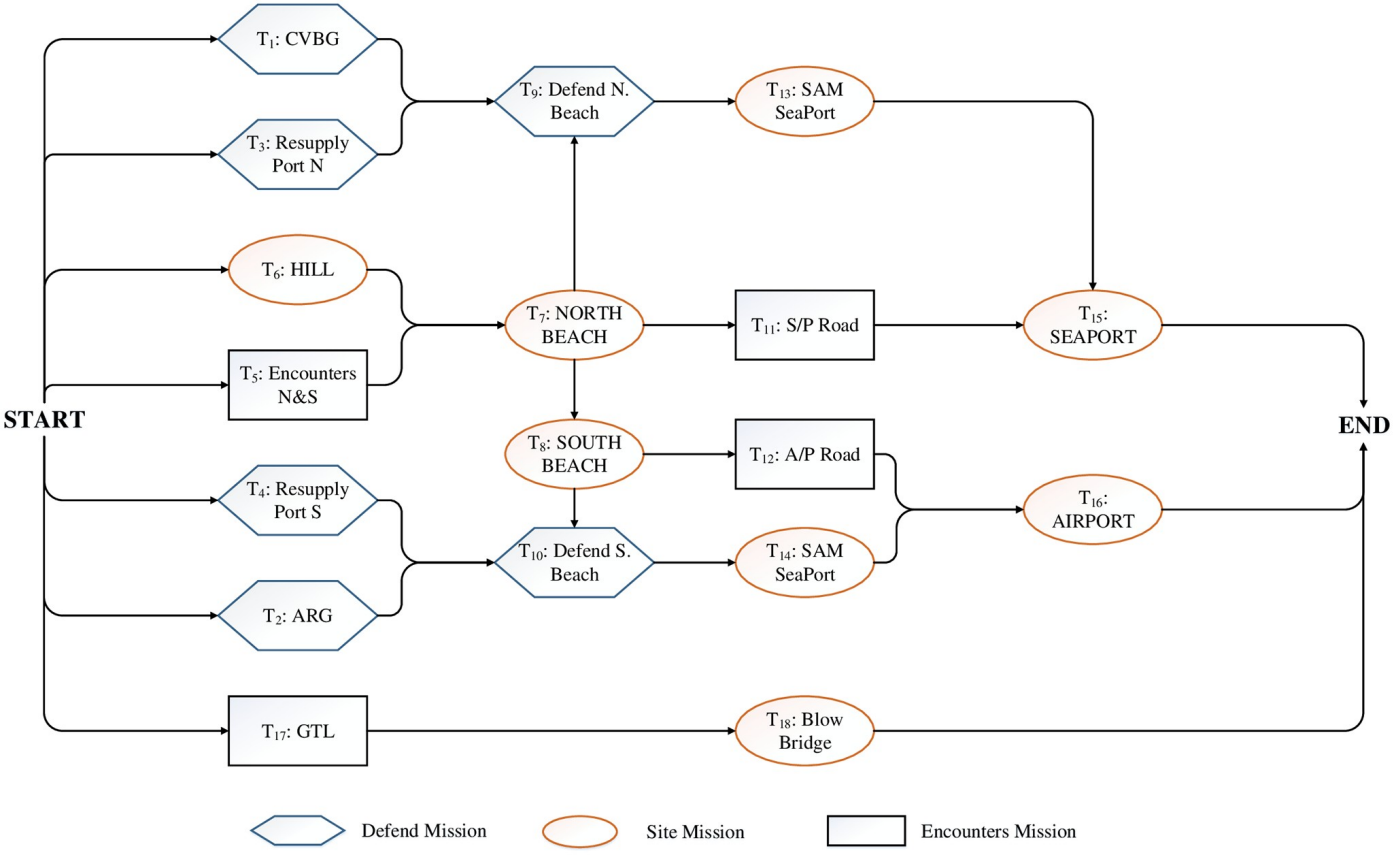
Network diagram of scenario task for simulation example.

In this paper, based on Experiment 7 [66] in the template library A2C2 of US military combat experiment scene, the MOOCTS model established in this paper is solved and analyzed. Experiment 7 in A2C2 was a joint landing operation, its combat goal is to occupy airports and ports and clearing the way for subsequent landing troops. There are a total of 18 combat tasks (*i* = 1, 2, …, 18), the task network diagram is shown in Fig 7.

There are 20 combat clusters (*k* = 1, 2, …, 20), represented by the number *P*1 − *P*20, there are 8 combat capabilities in this operation, namely AAW, ASUW, ASW, GASLT, FIRE, ARM, MINE and DES. Due to the large amount of data in the support information **S1 Excel**, we enumerate the combat capability of each combat cluster and the cluster capability data required for completing various combat tasks in detail. In addition, according to different combat missions, three groups of required times are set for comparative experiments. The comparison time data are shown in Table 3.

**Table 3:** The comparison time data of three groups of required times set for comparative experiments.

Instance	T1	T2	T3	T4	T5	T6	T7	T8	T9	T10	T11	T12	T13	T14	T15	T16	T17	T18
PT1	30	30	10	10	10	10	10	10	10	10	10	10	20	20	15	15	10	20
PT2	35	35	15	15	15	15	15	15	15	15	15	15	25	25	20	20	15	25
PT3	40	40	20	20	20	20	20	20	20	20	20	20	30	30	25	25	20	30

PT1, PT2 and PT3 respectively represent Processing Time1, Processing Time2 and Processing Time3. The three different sets of comparative data will change the scale of the comparative experiment.

The combat task durations (PT1, PT2, PT3) corresponding to three groups of combat missions with different intensities are set as the comparison conditions of the algorithm simulation results. $PT1,PT2,PT3=pt_{ij}^{k}(2)$, let $pt_{ij}^{k}(1)=pt_{ij}^{k}(2)(1-rand(1))$, $pt_{ij}^{k}(3)=pt_{ij}^{k}(2)\cdot (1+rand(1))$, where *rand*(1) is a function that generates evenly distributed random numbers between 0 and 1.

The combat flexible time is set as follows: in order to ensure sufficient combat coordination time, the SOUTH BEACH mission (T8) can only start 5 hours after the start of the NORTH BEACH mission (T7) at the earliest and must start 2 hours before the end of the T7 mission at the latest, that is $d_{7,8}^{\max}=5,d_{7,8}^{\min}=2$, similarly, let $d_{17,18}^{\max}=100,d_{17,18}^{\min}=85$.

### 5.2 Comparison algorithm

In this paper, four comparison algorithms are compared with the MOIBA/D algorithms, that is NSGA-II, MOEA/d, MOPSO and MOBA algorithm. The MOCCTS model is incorporated into the solution frameworks of the five algorithms, and the population coding mode of the comparative algorithm was consistent with the MOIBA/D algorithm. The algorithm initialization parameters were set as shown in Table 4.

**Table 4:** Parameter settings for 5 algorithms.

Algorithm	Parameter setting
NSGA-II	Population size: *N* = 1000, Mating pool size: *MP* = 600, Termination algebra: *iter*_max_ = 5000, Crossover probability: *Pc* = 0.8, Variation probability: *Pm* = 0.2, Crossover distribution index: *η*_*c*_ = 1, Variation distribution index: *η*_*m*_ = 1;
MOEA/D	Weight vector number: *N* = 1000, Neighbor vector number: *T* = 20, Termination algebra: *iter*_max_ = 5000, Crossover distribution index: *η*_*c*_ = 1, Variation distribution index: *η*_*m*_ = 1;
MOPSO	Weight vector number: *N* = 1000, Neighbor vector number: *T* = 20, Termination algebra: *iter*_max_ = 5000, Crossover distribution index: *η*_*c*_ = 1, Variation distribution index: *η*_*m*_ = 1;
MOBA & MOIBA/AD	population size *N* = 1000, maximum iteration number *iter*_max_ = 5000, initial pulse loudness *A*^0^ = 0, initial pulse emission rate *r*^0^ = 0, pulse loudness change rate and pulse emission rate change rate *α* = 0.9, *γ* = 0.05, pulse initial frequency *f*_0_ = 0, maximum and minimum pulse frequency *f*_*max*_ = 10, *f*_*min*_ = 0, individual initial velocity *v*_0_ = 0, maximum and minimum individual velocity disturbance coefficient *ω*_max_ = 1.2, *ω*_min_ = 0.1, amplitude of individual position disturbance function *A*_*m*_ = 2.

### 5.3 Performance metrics

Due to the complexity of the model and the unknown results, this paper selects the following four indicators as the performance evaluation indicators of the algorithm.

(1) Hypervolume (HV) [67]. The calculation formula is as follows:
  $$\begin{array}{c} HV=\lambda \left({⋃}_{i=1}^{\left|S\right|}v_{i}\right) \end{array}$$ (25)
  where λ is the Lebesgue measure, *v*_*i*_ is the hypervolume composed of the reference point *Z* and the non-dominated individual *Pop*_*i*_(*iter*_max_), and *S* is the non-dominated solution set. The calculation process does not need to know the Pareto optimal frontier, so it is very practical. Among many evaluation indicators, its monotonicity is good, and the larger the value, the better the convergence and distribution of the algorithm.
(2) Inverted generational distance (IGD) [68]. The calculation formula is as follows:
  $$\begin{array}{c} IGD=\sum_{\bar{j}\in PF^{*}}{d^{\prime }}_{\bar{j}}/n \end{array}$$ (26)
  where $d_{\bar{j}}^{\prime }=\min_{\bar{i}\in Pop\left(iter_{\max}\right)}|\bar{j}-\bar{i}|$, represents the minimum euclidean distance from a point on the optimal Pareto front to an individual in the final solution set. IGD can not only reflect that convergence of multi-objective evolutionary algorithm, but also reflect the distribution and universality of the solution set. The smaller the value is, the better the convergence, distribution and universality of the algorithm will be.
(3) Comprehensive performance evaluation index (MSS). This indicator is a further expansion of the statistical distribution of IGD indicators, and the calculation formula as:
  $$\begin{array}{c} MSS=\frac{1}{M}\sum_{m=1}^{M}\frac{I_{m}-\mu_{m}}{\delta_{m}} \end{array}$$ (27)
  where *M* is the number of optimization objectives, representing the IGD value of the evaluation algorithm on the *m*^*th*^ optimization objective, and *μ*_*m*_, *δ*_*m*_ is the mean and variance of the IGD value of the evaluation algorithm on the *m*^*th*^ optimization objective. Its value can not only reflect the convergence, distribution and universality of the algorithm reflected by IGD, but also reflect the statistical law of the final solution set.
(4) Number of dominated solutions. The index counts the number of dominated solutions in the final solution set of MOCTS models solved by different algorithms, and the numerical results obtained are used to express the performance of the algorithms in solving the model. Obviously, the more dominant solutions, the better the performance of the algorithm will be.

### 5.4 Result analysis

First of all, in order to avoid the influence of random error on the experimental results, in three calculation examples, five algorithms were run independently for 20 times. The average value, best value and worst value obtained by statistics are listed in the table below. The bold part was the optimal value of the probability degree of operational coordinative time under various indexes.

As shown in Table 5, the results of MOIBA/AD algorithm in different numerical examples are generally better than the four comparison algorithms. Although the optimal values are the same as those of NSGA-II algorithm and MOEA/D algorithm when solving numerical Instances 1 and 2, MOIBA/AD algorithm is obviously more reliable from the point of view of mean value and worst value. With the expansion of numerical instances, the advantages of MOIBA/AD algorithm gradually appear. In order to intuitively reflect the scheduling results of the five algorithms on the operational coordinative time, we draw the Gantt chart of the scheduling results of the five algorithms in the operational coordinative time.

**Table 5:** Comparison of simulation results of all algorithms.

Instance	Algorithm	Average value	Best value	Worst value
PT1	NSGA-II	**(158,166,174)**	**(150,160,170)**	(168,176,184)
	MOEA/D	(161,165,172)	**(150,160,170)**	**(162,170,181)**
	MOPSO	(162,166,177)	(158,166,174)	(166,177,186)
	MOBA	(164,170,179)	(158,166,174)	(164,172,184)
	MOIBA/AD	**(158,166,174)**	**(150,160,170)**	**(162,170,181)**
PT2	NSGA-II	(230,246,254)	**(220,230,240)**	**(232,240,251)**
	MOEA/D	**(228,236,244)**	**(220,230,240)**	(243,255,269)
	MOPSO	(232,249,258)	(228,239,251)	(242,256,271)
	MOBA	(234,251,266)	(229,238,247)	(240,250,261)
	MOIBA/AD	**(228,236,244)**	**(220,230,240)**	**(232,240,251)**
PT3	NSGA-II	(300,313,331)	(297,306,316)	(309,319,329)
	MOEA/D	(305,311,336)	(296,307,319)	(306,318,326)
	MOPSO	(306,313,334)	(297,310,318)	(305,313,326)
	MOBA	(301,311,330)	(299,308,317)	(303,311,321)
	MOIBA/AD	**(298,306,314)**	**(290,300,310)**	**(302,310,321)**

**Fig 8:**
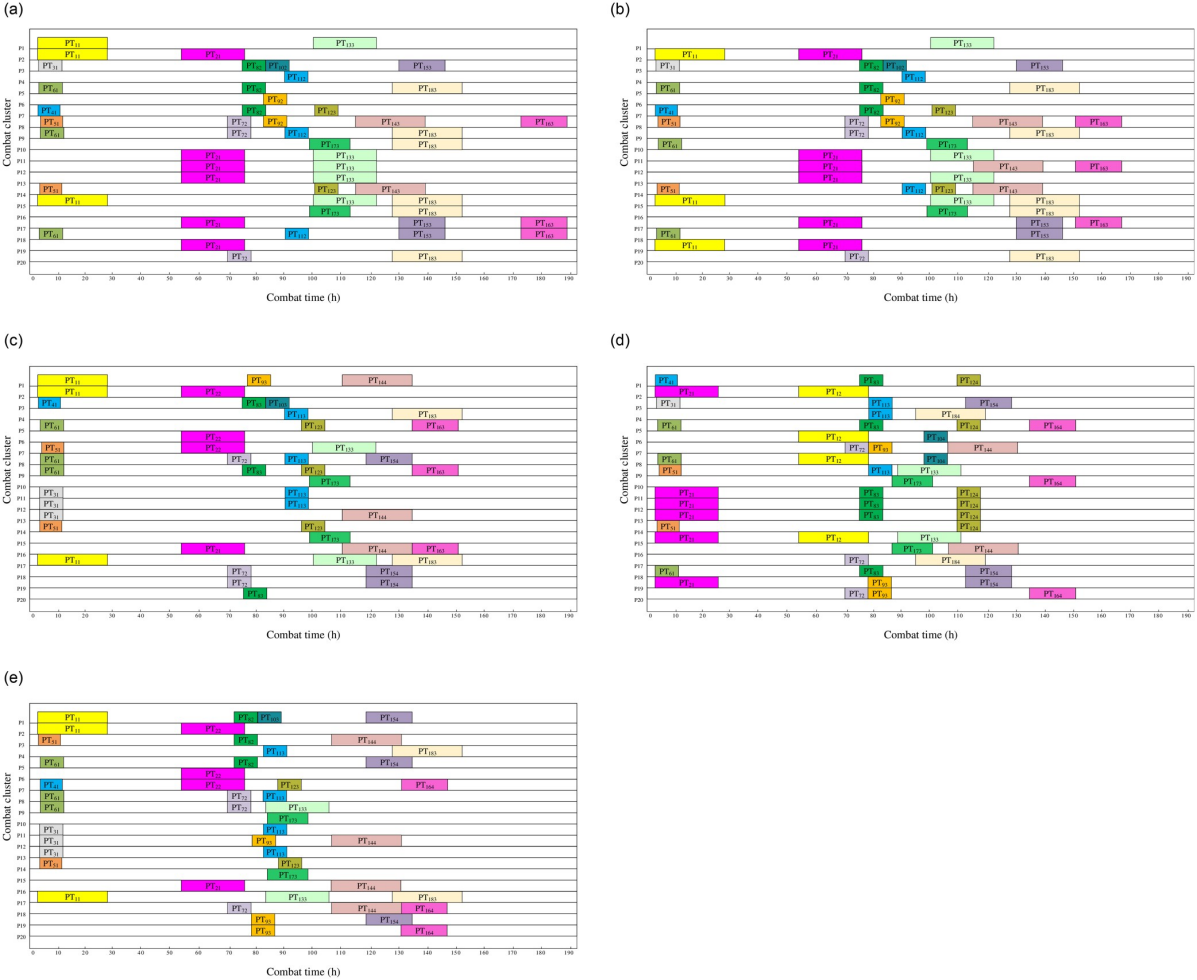
Gantt chart of operational coordination time planning results of five algorithms under simulation background.

In Fig 8, the durations of the same combat mission is indicated with the same color. It can be seen from the collaborative scheduling results that the scheduling time of MOIBA/AD algorithms is the least, while that of MOBA algorithms is the most, and the scheduling time of the other three algorithms is similar. Therefore, under the framework of MOIBA/AD algorithm, the performance of solving MOCCTS model is optimal.

The comparison results of HV, IGD, MSS and the number of dominated solutions indexes obtained by simulation are shown in Table 6. It can be concluded that, on the whole, the performance of MOIBA/AD is superior to that of the comparison algorithm, and the advantages of MOIBA/AD are more obvious when the scale of the MOOCTS model is expanded. Specifically, on the index HV and the index MSS, the performance of MOIBA/AD is better than that of the comparison algorithm, and the effect of the algorithm is still excellent after the model scale is expanded. On the indicator IGD, the algorithm still maintains a good effect on the whole, except that the performance of the algorithm is similar to that of NSGA-II after the scale is enlarged. It can also be concluded that when NSGA-II solves large-scale multi-objective problems with sufficient number of iterations, the algorithm has excellent performance. In summary, MOIBA/AD obtains five optimal values in six groups of data under four indicators, which is very effective in solving the MOOCTS model.

**Table 6:** The values of HV, IGD, MSS and the number of non-dominated solutions corresponding to each of the comparison algorithms in solving three different-scale cooperative time scheduling problems.

Parameter	Instance	MOBA	MOPSO	MOEA/D	NSGA-II	MOIBA/AD
**HV**	PT1	0.1143−(0.0916−)	0.4892 +(0.3793 +)	0.1528 −(0.1134 +)	0.1662 (0.1091)	**0.5112** +(**0.3823** +)
	PT2	0.4861−(0.4555−)	0.5392 +(0.4491 +)	0.4966 −(0.4135 −)	0.5026 (0.4756)	**0.5613** +(**0.4624** +)
	PT3	0.4987−(0.4235−)	0.5045 +(0.4589 +)	0.4817 −(0.4145 −)	0.5137 (0.4512)	**0.5823** +(**0.4714** +)
**IGD**	PT1	1.323−(0.967−)	1.256 −(1.002 −)	0.088 +(0.061 +)	0.553 (0.144)	**0.037** +(**0.025** +)
	PT2	0.084−(0.012−)	0.107 −(0.044 −)	0.116 −(0.192 −)	**0.053** (**0.008**)	**0.054** =(**0.013** =)
	PT3	1.023−(0.614−)	1.151 −(1.101 −)	0.188 +(0.161 +)	0.603 (0.214)	**0.437** +(**0.326** +)
**MSS**	PT1	10.231+	−1.761 +	1.433 +	23.899	−**6.661** +
	PT2	17.891+	−**18.231** +	6.963 +	24.691	−11.781 +
	PT3	17.232+	11.766 +	6.413 +	25.781	−**16.002** +
**No**. **of dominated solution**	PT1	311−	321−	320−	**348**	**347**=
	PT2	291+	311+	330+	290	**462**+
	PT3	280−	348−	**383**+	360	**382**+
+/−/≈		4/8/0(4/8/0)	7/5/0(7/5/0)	7/5/0(8/4/0)		10/0/2(10/0/2)

“+ /−/ =” represents that the test algorithm is superior, equivalent and inferior to the comparison algorithm, respectively. The optimal value of each index data is expressed in bold, with the data outside brackets as the optimal value of the index and the data in brackets as the average value of the index.

**Fig 9:**
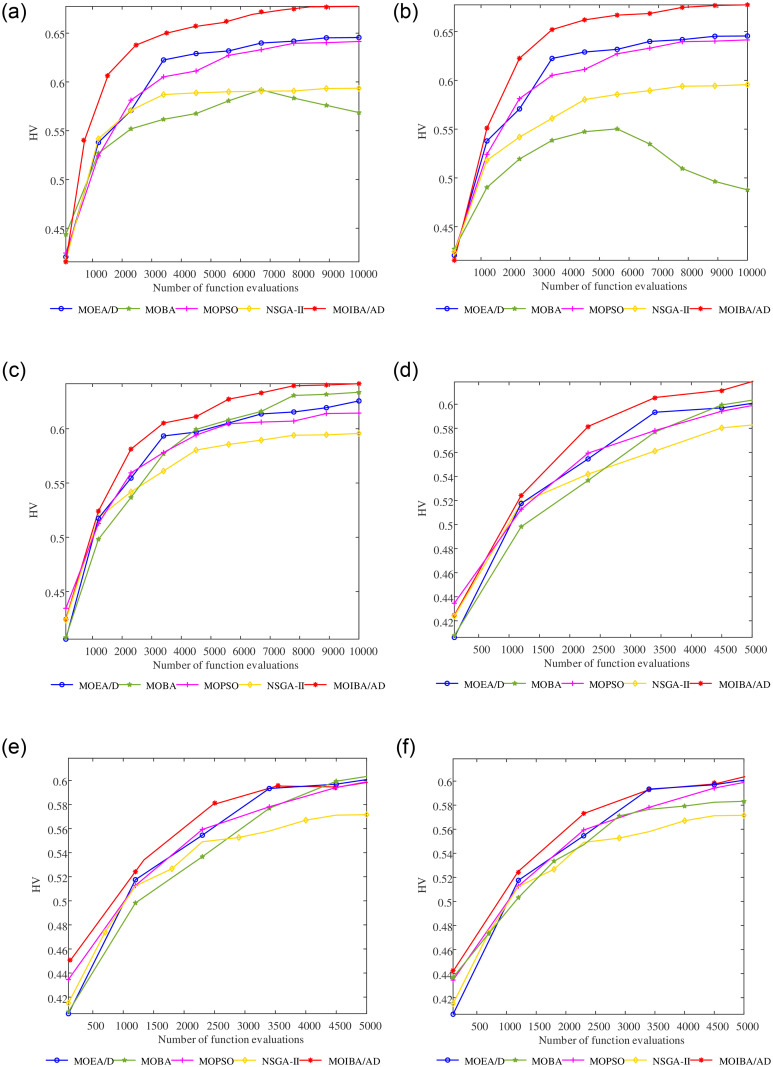
Average convergence curves of the HV indexes on the model of three different scales, obtained by a large-scale model with 10000 iterations (a)-(c), a small-scale model with 5000 iterations (d)-(f).

**Fig 10:**
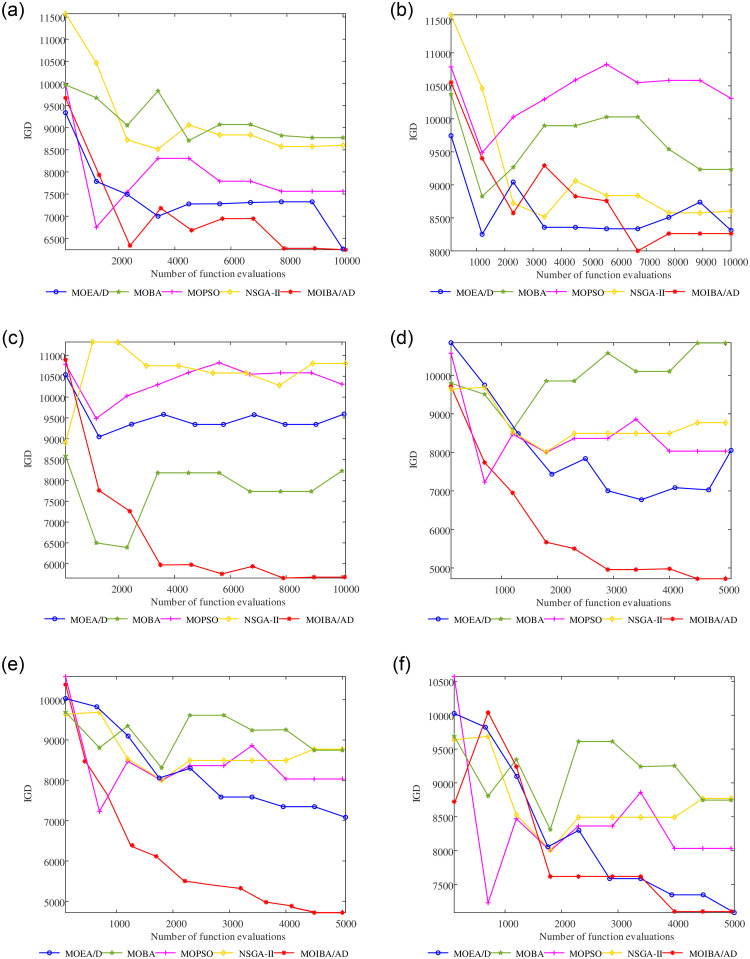
Average convergence curves of the IGD indexes on the model of three different scales, obtained by a large-scale model with 10000 iterations (a)-(c), a small-scale model with 5000 iterations (d)-(f).

The average convergence curves of indicators HV and IGD on three different scale models are shown in Figs 9 and 10. It can be seen that when solving the MOOCTS model with a small scale, the performance of the four comparison algorithms is unstable. The convergence rates of MOBA, NSGA-II and MOEA/D in the HV index are too slow, and the index still fails to converge when the maximum number of iterations is reached. The convergence rates of MOBA, NSGA-II and MOPSO in the IGD index are too slow, which are more than 2000 generations later than the comparison algorithm. However, MOIBA/AD shows good index convergence when solving the MOOCTS model, regardless of the large or small scale.

**Fig 11:**
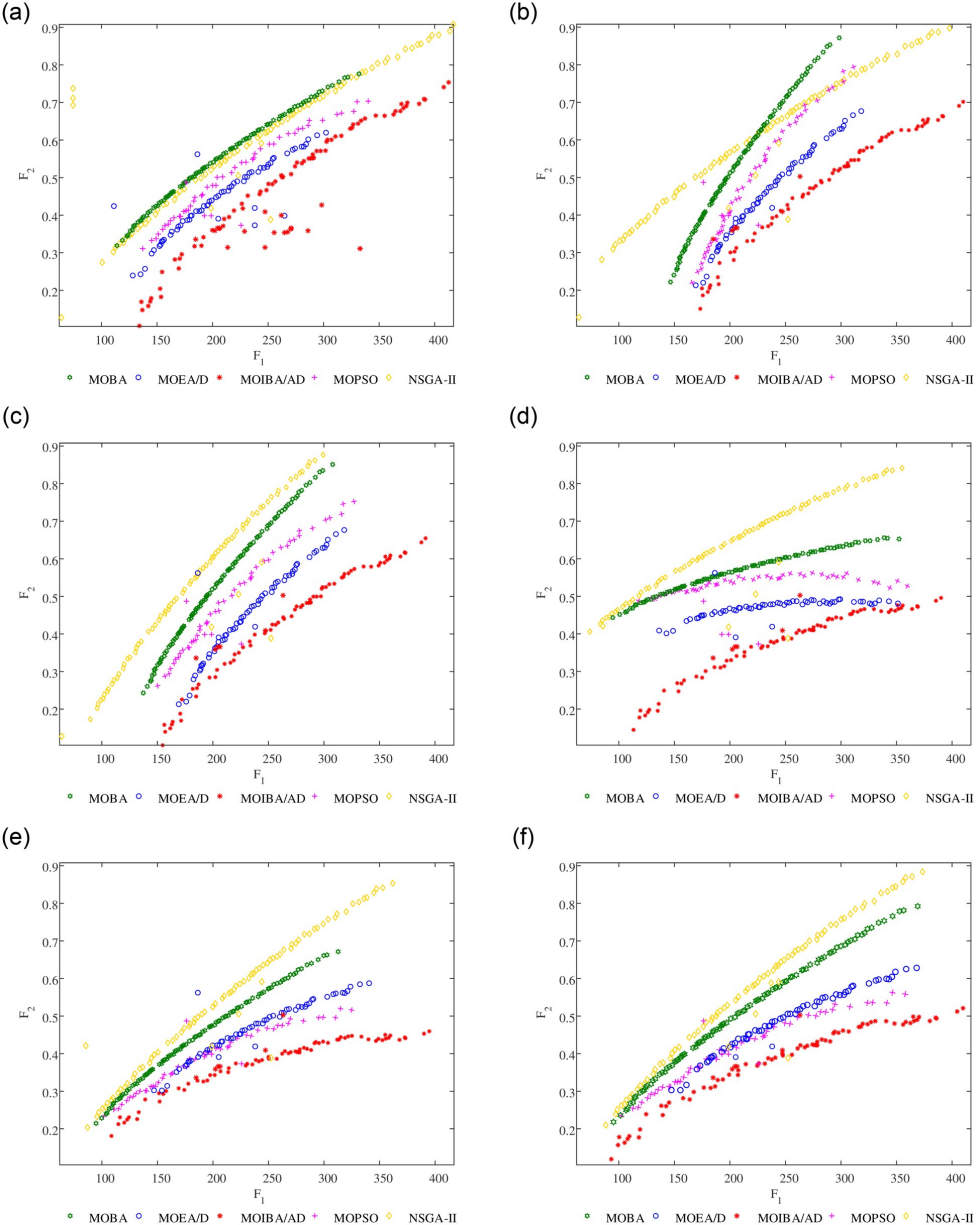
PF of optimization objective and illustration of parallel coordinates of the non-dominated fronts on three different scale ECMJTA models, obtained by small-scale model with 5000 iterations (a)-(c), large-scale model with 10000 iterations (d)-(f).

In the simulation process, when the five algorithms solve the MOOCTS models of three different sizes, PF of optimization objective and illustration of parallel coordinates of the non-dominated fronts on 3 different scale MOOCTS models are shown in Fig 11. It can be seen that the diversity and convergence of MOIBA/AD are better than those of MOBA, NSGA-II, MOEA/D and MOPSO when solving the small-scale MOOCTS model. NSGA-II has poor diversity and convergence, MOEA/D has good diversity but poor convergence, and MOPSO has poor diversity but poor convergence; when solving large-scale MOOCTS models, the diversity and convergence of MOIBA/AD are better than those of MOBA, NSGA-II, MOEA/D and MOPSO. However, the diversity and convergence of the remaining comparison algorithms are greatly improved.

## 6 Conclusion and future work

In modern war, scheduling of operational coordinative time is one of the most important tasks in operational coordination. In this study, in order to solve this problem, we established the multi-objective operational cooperative time scheduling (MOCCTS) model. This problem is a complex multi-objective combinatorial optimization problem with the characteristics of multi-decision variables and multi-objective functions.In this paper, we propose a MOIBA/AD algorithm to solve this problem. This algorithm improves the MOEA/D by changing the aggregate function strategy used by MOEA/D to the strategy using angle space decomposition, thus greatly reducing the computational complexity. Moreover, the population renewal strategy is improved through the optimization of the IBA, considering that the IBA is not easy to enter the local optimum, the population renewal effect of the algorithm is effectively improved. Finally, MOIBA/AD is compared with four multi-objective evolutionary algorithms such as MOBA, NSGA-II, MOEA/D and MOPSO to solve three MOOCTS models with different scales. The comparison of performance indexes indicates that MOIBA/AD has good population convergence and distribution.

In conclusion, MOIBA/AD can effectively and efficiently solve the MOOCTS model of different sizes, and provide decision recommendations for the commander in the combat process.The MOOCTS problem proposed in this paper is actually an extension of the multi-task time scheduling problem, which only schedules the cooperative time in the course of combat. However, we do not consider the scheduling the remaining operational factors (i.e. operational resources, electromagnetic spectrum.) in a real and dynamic environment. Besides, in the process of solving the cooperative time sequence of combat missions, the method proposed in this paper first needs to analyze the time network among combat tasks. When the scale of combat mission expands, this step will produce great time complexity, which will greatly affect the practical application.

In the further, on the one hand, we need to further expand the MOOCTS model, take into account the factors such as combat support resource constraints and electromagnetic spectrum resource constraints, and add the comprehensive analysis of node factors on the basis of the directed graph processing of combat sequence, so that the model is closer to the actual combat. On the other hand, we will explore the algorithm of solving large-scale directed graphs and the processing multiple directed graphs in parallel in the model initialization, which can be applied to large-scale operations. In addition, the role of MOIBA/AD in solving other combinatorial optimization problems is also studied, and it is applied to more fields and problems.

## Supporting information

S1 Data Simulation data sheet. Combat capability and cluster capability simulation data sheet of each combat cluster required for completing various combat tasks.
(XLSX)

S1 Appendix Simulation process of MOIBA/AD algorithm. (PDF)
