# Supplementary material for: A multi-objective scheduling method for operational coordination time using improved triangular fuzzy number representation
Source: PLoS One. 2021 Jun 9;16(6):e0252293. doi: 10.1371/journal.pone.0252293 (PMC8189490; doi:10.1371/journal.pone.0252293)
Supplement: S1 Appendix — (PDF) [file pone.0252293.s002.pdf]

S1 Appendix. Simulation process of MOIBA/AD algorithm. In this paper, the simulation environment is MATLAB 2020b and the simulation platform is PlatEMO 3.0 (`https://github.com/BIMK/PlatEMO`, cite as: 'Tian Y, Cheng R, Zhang X, Jin Y. PlatEMO: A MATLAB platform for evolutionary multi-objective optimization [educational forum]. IEEE Computational Intelligence Magazine. 2017;12(4):73–87.'). Most of the multi-objective evolutionary algorithms are integrated in this platform. We embed the algorithm 1 code proposed in this paper into the platform to compare the performance of the algorithm in the simulation process.
